# Would You Buy on Impulse During E-Commerce Digital Human Avatar Live Shopping? DHA Characteristics and Consumer Experience

**DOI:** 10.3390/bs16101961

**Published:** 2026-10-21

**Authors:** Siying Mao, Euitay Jung

**Affiliations:** 1 Major of Visual Design, Graduate School, Hanyang University, Seoul 04763, Republic of Korea; maosiying@hanyang.ac.kr; 2 Department of Communication Design, College of Design, Hanyang University ERICA, Ansan 15588, Republic of Korea

**Keywords:** e-commerce digital human avatar (EC-DHA), impulse buying intention, EC-DHA characteristics, consumer experience, S-O-R model, mixed-methods research

## Abstract

Digital human avatars (DHAs) are emerging as a new type of intelligent shopping agent in livestream e-commerce; however, existing research has not fully explained how their characteristics influence impulse buying through consumer experience mechanisms. Drawing on the Stimulus–Organism–Response (S-O-R) framework, this study conceptualizes the interactivity, empathy, immersion, and vividness of e-commerce digital human avatars (EC-DHAs) as external stimuli and visual appeal, consumer-perceived arousal, and consumer engagement as internal experiential mechanisms, and examines their relationships with consumer impulse buying intentions. A sequential explanatory mixed-methods design was adopted. In the quantitative phase, purposive sampling was employed, and all participants were exposed to the same standardized EC-DHA livestream stimulus. Based on 467 valid questionnaires, partial least squares structural equation modeling (PLS-SEM) was used to examine the structural relationships, followed by artificial neural network (ANN) analysis to assess the relative predictive importance of key factors. In the qualitative phase, purposive sampling was used to recruit 21 participants for semi-structured interviews, and a grounded theory-informed coding procedure was applied to contextualize and further interpret the quantitative findings. The results show that interactivity and vividness are significantly and directly associated with consumer impulse buying intentions, whereas the direct relationships of empathy and immersion with impulse buying intention are not significant. Instead, empathy and immersion are primarily indirectly associated with impulse buying intention through experiential mechanisms, including visual appeal, consumer-perceived arousal, and consumer engagement, underscoring the important role of consumer experience in linking EC-DHA characteristics to impulse buying intention. The ANN results further identify visual appeal, vividness, and interactivity as key predictors of impulse buying intention. Overall, different EC-DHA characteristics exhibit distinct direct and indirect pathways to consumer impulse buying intentions. These findings clarify the role of consumer experience mechanisms in these relationships and provide practical implications for the visual presentation, interactive feedback, emotional expression, and consumer guidance of EC-DHAs in livestream e-commerce.

## 1. Introduction

As artificial intelligence technologies become increasingly integrated into livestream e-commerce, online shopping environments are evolving from product-centered displays toward more interactive and experience-oriented consumption settings (Dwivedi et al., 2021; Verhoef et al., 2021). E-commerce digital human avatars (EC-DHAs) are emerging as a new type of interactive avatar that connects consumers, products, and purchase decisions on digital platforms. Unlike conventional pre-scripted virtual avatars, EC-DHAs are defined not merely by their anthropomorphic appearance, but also by AI-enabled capabilities for real-time interaction, information response, and purchase guidance. They can be conceptualized as AI-driven virtual shopping avatars with human-like appearances and interactive capabilities that support product demonstrations, real-time responses, emotional expression, and purchase guidance in livestreaming environments (Yuan et al., 2025). Accordingly, EC-DHAs reshape how consumers access product information by integrating anthropomorphic presentation, real-time responsiveness, and personalized interaction, thereby creating a dynamic interactive experience distinct from traditional e-commerce interfaces (Gao et al., 2023; C. Li & Huang, 2024; Wen & Li, 2025). Against this background, examining how EC-DHAs are associated with consumers’ psychological responses and purchase decisions from a consumer experience perspective is both theoretically and practically important.

Against this background, this study proposes that EC-DHAs may shape consumer experience through interactivity, empathy, immersion, and vividness, thereby stimulating impulse buying intention. As AI-driven intelligent interactive avatars, EC-DHAs perform multiple functions in live-streaming e-commerce, including shopping guidance, product recommendation, emotional expression, and product explanation. Their influence on consumers differs from that of traditional human streamers, whose effects often depend on personal charisma or social attractiveness. Instead, EC-DHAs are more likely to affect consumers through the combined effects of algorithmic interaction, visual presentation, and experiential arousal. Previous studies have shown that interactivity, visual presentation, and social presence in live-streaming e-commerce influence consumer engagement and purchase intention (Sun et al., 2019), while impulse buying is generally driven by immediate stimuli, emotional arousal, and online shopping experience (Lee & Chen, 2021; Verhagen & Van Dolen, 2011). However, in the context of rapid advances in artificial intelligence, limited research has systematically explained how EC-DHA characteristics are transformed into visual appeal, perceived arousal, and consumer engagement, or how these experiential mechanisms further promote impulse buying intention.

Although previous studies have examined virtual streamers, digital humans, and AI-driven virtual streamers and identified interactivity, anthropomorphism, social presence, trust, and experiential value as important factors associated with consumer purchase responses (Gao et al., 2023; C. Li & Huang, 2024; Wu et al., 2023), three issues remain insufficiently addressed in the EC-DHA context. First, the conceptual boundaries surrounding EC-DHAs remain unclear. Existing studies use terms such as virtual streamer, digital human, AI-enabled virtual streamer, and avatar to describe virtual entities in livestream e-commerce. However, EC-DHAs combine anthropomorphic presentation, AI-driven interaction, and real-time shopping assistance, and their theoretical positioning therefore requires further clarification (Gao et al., 2023; L. Wang et al., 2026). Second, prior research on consumer experiential mechanisms has primarily focused on variables such as social presence, experiential value, cognitive trust, and emotional arousal (M. Li et al., 2022; Wu et al., 2023), whereas visual appeal, perceived arousal, and consumer engagement have rarely been integrated within a unified EC-DHA research framework. Third, it remains unclear whether different EC-DHA characteristics influence impulse buying intention through similar or distinct pathways. In particular, the extent to which empathy and immersion exert direct effects, rather than operating indirectly through visual appeal, perceived arousal, and consumer engagement, has not been systematically examined. Accordingly, this study develops an “EC-DHA characteristics–consumer experiential states–impulse buying intention” model grounded in the S-O-R framework to clarify the differentiated pathways through which distinct EC-DHA characteristics are associated with consumers’ impulse buying intention.

Building on previous applications of the S-O-R framework in EC-DHA research, this study develops and empirically tests an “EC-DHA characteristics–consumer experiential states–impulse buying intention” model centered on consumer experience. Interactivity, empathy, immersion, and vividness are conceptualized as external stimuli; visual appeal, consumer-perceived arousal, and consumer engagement as internal experiential states; and consumer impulse buying intention as the final behavioral response. The S-O-R framework posits that external environmental stimuli influence behavioral responses through individuals’ internal psychological states, making it particularly suitable for explaining consumer experience and purchase intention in EC-DHA livestreaming contexts (Arora, 1982; Lee & Chen, 2021). Methodologically, this study adopts a sequential explanatory mixed-methods design. First, the research variables and hypothesized relationships are established through a review of the relevant literature and theoretical reasoning. Second, PLS-SEM is used to examine the linear relationships among the study variables (Hair et al., 2019). Third, ANN analysis is employed to identify potential nonlinear predictive relationships among the variables. Finally, semi-structured interviews and grounded theory-informed coding procedures are used to contextualize and further explain the quantitative findings (Corbin et al., 2008; Venkatesh et al., 2013). This qualitative phase complements the PLS-SEM and ANN analyses by providing contextual explanations of the observed relationships, thereby offering a more systematic understanding of how EC-DHA characteristics influence impulse buying intention through consumer experiential mechanisms.

Based on the research gaps identified above, this study makes three main contributions. First, it provides a contextual refinement of the Organism component of the S-O-R framework rather than merely adding additional consumer response variables. Traditional S-O-R research generally conceptualizes the organism as the internal cognitive and affective states that arise in response to external stimuli. In the EC-DHA livestreaming context, however, consumer experience encompasses visual evaluations of the digital human and interface, affective activation triggered by interaction and the livestream atmosphere, and engagement that develops through continued viewing and interaction. Accordingly, this study conceptualizes visual appeal, consumer-perceived arousal, and consumer engagement as three complementary experiential dimensions corresponding to visual evaluation, affective activation, and participatory involvement, respectively. It then examines how EC-DHA characteristics are associated with impulse buying intention through these differentiated experiential pathways. Second, this study integrates PLS-SEM, ANN, and grounded theory-informed qualitative analysis to examine linear relationships among the variables, identify potential nonlinear predictive relationships involving key factors, and use interview data to further contextualize consumers’ psychological responses and behavioral motivations in livestream shopping settings (Hair et al., 2019; Venkatesh et al., 2013). Third, this study provides practical implications for the design and operation of EC-DHAs. The findings suggest that platforms should not focus solely on algorithmic recommendations and functional presentation but should also optimize interaction styles, emotional expression, visual presentation, and the overall livestream experience to enhance consumer experience and inform the design of strategies that encourage consumer engagement and purchase intention. Given the model’s limited explanatory power for consumer experience variables and impulse buying intention, these theoretical and practical implications are intended primarily to clarify patterns of association among the variables rather than to suggest that EC-DHA characteristics can fully explain or determine consumer behavior.

## 2. Literature Review and Theoretical Model

### 2.1. Conceptualizing E-Commerce Digital Human Avatar in Live-Streaming Commerce

A digital human avatar is an AI-driven intelligent digital avatar in a virtual environment. It typically possesses an anthropomorphic appearance, natural language interaction, emotional expression, and autonomous capabilities for perception, reasoning, and decision-making. Such avatars can proactively perform tasks in response to user needs and environmental changes, while supporting continuous and natural human–computer interaction through visual, speech, and behavioral feedback (Park et al., 2023; D. Wang et al., 2024). In e-commerce, these virtual avatars are further embodied as e-commerce digital human avatars (EC-DHAs) and are widely used in live-streaming sales, product recommendation, brand communication, and shopping companionship. In these contexts, they simulate the roles of human streamers, sales consultants, or brand endorsers (Wen & Li, 2025). Unlike general recommendation systems or intelligent customer service avatars, EC-DHAs do more than transmit information. They enhance consumer engagement and purchase desire through product explanation, interactive demonstration, visual presentation, and emotional expression, thereby promoting impulse buying intention (C. Li & Huang, 2024).

**Figure 1:**
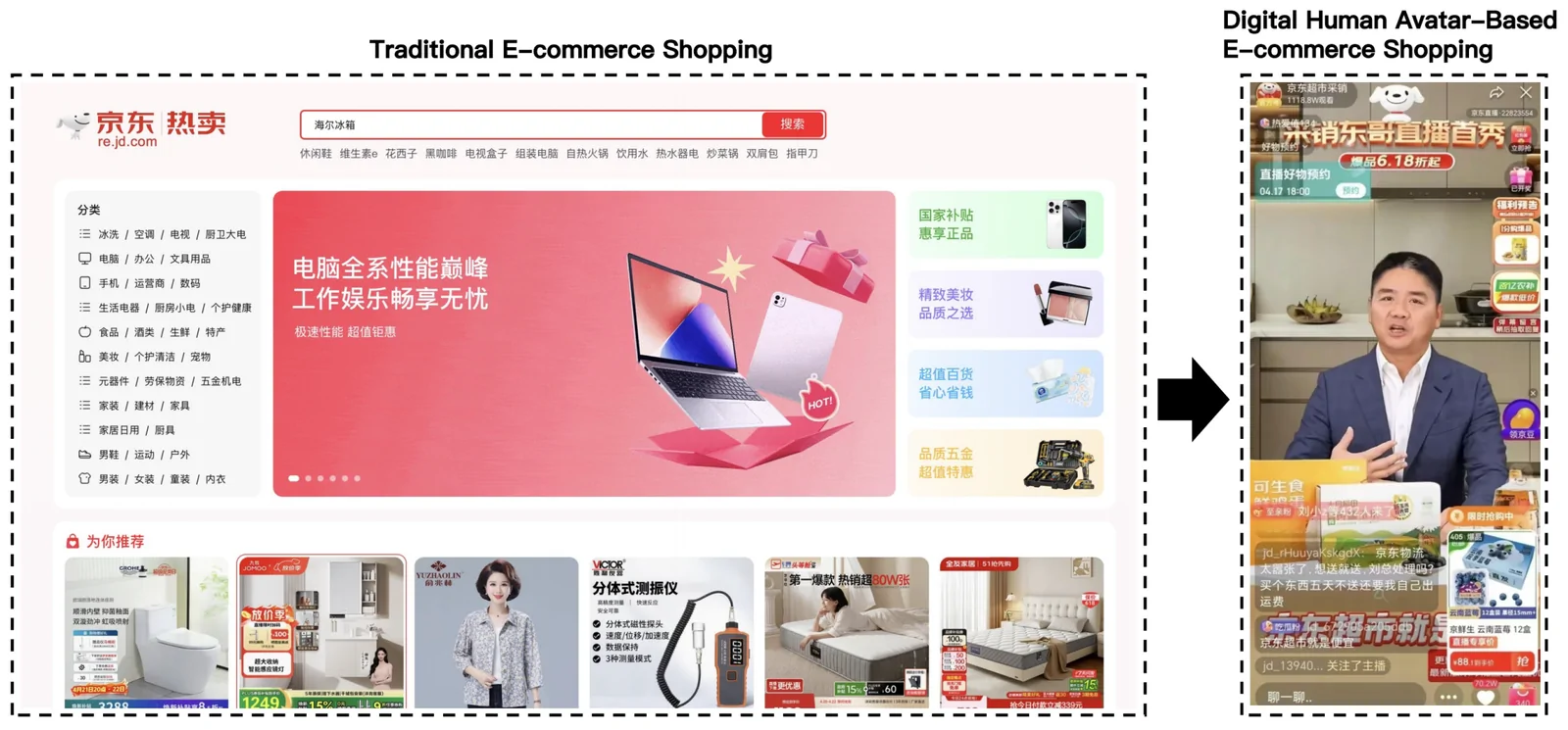
Comparison of JD.com’s traditional e-commerce shopping interface (left) and digital human avatar–based live-streaming e-commerce interface (right). Note: The Chinese text in the screenshots represents the original interface content of JD.com. In the traditional shopping interface, the Chinese labels indicate product categories, promotional information, and product recommendations. In the live-streaming interface, the Chinese text refers to promotional messages, product descriptions, and interactive shopping features. The original Chinese text is retained to preserve the authenticity of the platform interfaces.

In this study, a digital human avatar (DHA) is defined as an AI-powered virtual shopping guide embedded in e-commerce live-streaming environments. For example, “Caixiao Dongge AI Digital Human,” a virtual digital human modeled after Liu Qiangdong, the founder of JD.com, enables consumers not only to browse products and product information through traditional interfaces but also to engage in more interactive product presentations and purchase decision-making processes. Compared to traditional virtual shopping guides, however, EC-DHAs differ significantly in their interaction logic and communication functions (Figure 1). Traditional e-commerce shopping typically relies on menu-based navigation, product list recommendations, and consumer-initiated searches, with interaction mainly centered on interface-based information browsing and filtering. In contrast, e-commerce digital human avatars (EC-DHAs) are embodied virtual characters that dynamically communicate with consumers through language interaction, emotional expression, and scenario-based product presentation. They also support product recommendation, atmosphere creation, trust building, and purchase guidance. Therefore, EC-DHAs are not merely a new interactive medium connecting consumers and products; their core value lies in enhancing consumers’ shopping engagement and purchase intention through immersive experiences.

Although concepts related to digital humans overlap to some extent in the existing literature, they differ in their primary focus. A digital human avatar is a broad concept referring to a digitally represented virtual character with anthropomorphic appearance or behavioral features and may be controlled by humans, operated through predefined programs, or powered by artificial intelligence. In contrast, a virtual streamer emphasizes the role of a virtual character as a livestream host whose primary function is to present content and interact with audiences in real time through a virtual persona, although it is not necessarily autonomously driven by AI. A virtual influencer is mainly situated in social media and brand communication contexts, where its core functions include content dissemination, audience relationship building, and marketing influence. An AI-generated streamer, by comparison, emphasizes the use of AI technologies to generate content, language, or interactive behaviors, with particular attention to the degree of automation and technological generation. Building on these distinctions, the EC-DHA examined in this study represents a context-specific application of digital human technology in livestream e-commerce. Specifically, it refers to an AI-driven intelligent commercial avatar that uses an anthropomorphic digital character as an embodied interface and is capable of real-time interaction, product presentation, product recommendation, and shopping guidance during livestreaming. These concepts are therefore not mutually exclusive but instead emphasize different dimensions, including virtual representation, livestreaming roles, social influence functions, AI-based generation mechanisms, and e-commerce-related tasks.

A structured review of studies on EC-DHAs and virtual streamers identifies three major research gaps.

First, concepts such as digital human avatars, virtual streamers, virtual influencers, AI-generated streamers, and EC-DHAs differ in their emphasis on virtual representation, technological mechanisms, communication roles, and application contexts. However, their use in the existing literature remains partially overlapping and inconsistent. Previous studies have examined virtual streamers, EC-DHAs, AI-driven virtual streamers, and digital humans either separately or in comparison with human streamers, depending on the research context (Gao et al., 2023; Hu & Ma, 2023; Xu et al., 2025; Yan et al., 2025). Although these studies have explored the roles of virtual avatars in livestream e-commerce from different perspectives, the conceptual boundaries and functional distinctions among different types of virtual avatars remain insufficiently defined. Moreover, while prior research has identified key characteristics such as anthropomorphism, sociality, responsiveness, and interactivity, the theoretical boundaries and context-specific positioning of EC-DHAs, which integrate anthropomorphic representation, AI-driven interaction, and real-time shopping guidance, require further clarification.

Second, prior research has identified a range of psychological mechanisms through which EC-DHAs influence consumers, including social presence, telepresence, experiential value, perceived warmth and competence, cognitive trust, psychological contract, perceived value, attractiveness, and cognitive fluency (Gao et al., 2023; Gu et al., 2026; C. Li & Huang, 2024; H. Liu et al., 2025; Qin & Liu, 2025; Wu et al., 2023; Yao et al., 2024). However, these mechanisms have largely been examined in isolation, leaving the combined roles of visual, affective, and engagement-related experiences insufficiently understood. In particular, visual appeal, perceived arousal, and consumer engagement have rarely been incorporated simultaneously into a unified theoretical framework in EC-DHA research or examined as complementary dimensions of consumer experience.

Third, prior research has applied the S-O-R framework to EC-DHA contexts and begun to examine its relationship with impulse buying intention. For example, Wen and Li (2025) examined the relationships among digital human characteristics, emotional arousal, and purchase intention, whereas Zhou and Li (2025) conceptualized responsiveness, likability, professionalism, and anthropomorphism as stimuli; trust and flow experience as organismic states; and impulse buying intention as the behavioral response. However, existing studies have not sufficiently differentiated the direct and indirect pathways through which specific EC-DHA characteristics are associated with impulse buying intention. In particular, it remains unclear whether empathy and immersion are directly related to impulse buying intention or are primarily associated with it indirectly through experiential mechanisms such as visual appeal, perceived arousal, and consumer engagement. These relationships therefore require further empirical examination.

To strengthen the evidential foundation of the literature review, this study conducted a structured search of the Web of Science Core Collection and Scopus databases. The search covered publications from 2022 to 2026 and combined digital human-related terms (e.g., “digital human,” “virtual streamer,” “virtual anchor,” and “AI avatar”) with terms related to e-commerce and consumer behavior (e.g., “e-commerce,” “live-streaming commerce,” “purchase intention,” and “impulse buying”). Studies were included if they examined digital humans or comparable virtual intelligent avatars in e-commerce or consumer contexts and addressed consumer cognition, experience, or behavioral outcomes. Studies focused primarily on technical implementation or non-commercial contexts were excluded. Eligible studies were identified through title, abstract, and full-text screening and are summarized in Table 1.

In summary, although prior studies have examined virtual streamer characteristics and consumer responses from multiple perspectives, the underlying mechanisms remain fragmented. Drawing on the S-O-R framework, this study therefore investigates how interactivity, empathy, immersion, and vividness are associated with consumers’ impulse buying intention through visual appeal, perceived arousal, and consumer engagement.

### 2.2. Stimulus-Organism-Response Model

The stimulus–organism–response (S-O-R) theory originates from environmental psychology and explains how individuals respond behaviorally to external environmental stimuli (Mehrabian & Russell, 1974). It emphasizes the influence of external stimuli on individuals’ internal psychological states. The S-O-R model consists of three components: stimulus, organism, and response (Arora, 1982). Stimulus (S) refers to external factors that influence user behavior, whereas organism (O) represents internal psychological states, including users’ perceptual experiences. Response (R) refers to behavioral outcomes triggered by cognitive and emotional changes arising from the interaction between stimuli and the organism. These outcomes may take the form of either approach or avoidance behaviors (Sherman et al., 1997). Approach behaviors include positive actions such as exploration, continued presence, and participation, whereas avoidance behaviors involve opposite responses, such as reluctance to engage in positive actions (R. Zhang et al., 2024).

This theoretical mechanism was subsequently extended to online consumer environments. Drawing on the S-O-R framework, Eroglu et al. (2003) showed that external cues in online shopping environments shape consumers’ shopping responses through internal affective and cognitive states. This finding demonstrates the applicability of the S-O-R model to explaining the relationships among environmental stimuli, internal experiences, and behavioral responses in digital commercial contexts. The S-O-R model provides an appropriate theoretical framework for explaining how external stimuli influence behavioral responses through consumers’ internal experiences in e-commerce digital human avatar (EC-DHA) live-streaming contexts. For example, Zhou and Li (2025) examined the formation of consumers’ impulse buying intention in virtual AI streamer contexts using the S-O-R model. Their theoretical pathway conceptualized virtual streamers’ perceived responsiveness, likability, expertise, and anthropomorphism as stimuli; trust and flow experience as organismic states; and impulse buying intention as the response. C. Li and Huang (2024) further showed that the anthropomorphic characteristics of virtual streamers significantly influence consumers’ purchase intention through cognitive trust. This finding suggests that the observable features and social cues of digital humans affect consumer behavior by shaping consumers’ internal psychological perceptions. However, existing studies have mainly emphasized trust, flow, or purchase intention as core mechanisms. Less attention has been given to the roles of visual appeal, perceived arousal, and consumer engagement in linking EC-DHA characteristics to impulse buying intention from an experience design perspective.

Accordingly, this study applies the S-O-R model to the EC-DHA livestream e-commerce context to explain how EC-DHA-related stimuli are associated with impulse buying intention through consumers’ internal experiences. Although the S-O-R framework is broadly applicable, its flexibility may allow for diverse antecedents, psychological states, and behavioral outcomes to be retrospectively categorized as S, O, and R, thereby weakening theoretical constraints. To address this concern, the pathways examined in this study were not specified retrospectively based on the empirical results. Instead, the theoretical roles of the variables were defined a priori based on the EC-DHA context and relevant theoretical foundations. Specifically, the stimulus component (S) comprises interactivity, empathy, immersion, and vividness; the organism component (O) includes visual appeal, consumer-perceived arousal, and consumer engagement; and the response component (R) is represented by consumer impulse buying intention. Importantly, immersion in this study refers to consumers’ perceptions of the immersive characteristics of the EC-DHA livestream environment rather than to a subsequent internal psychological state. Therefore, the mediation results should be interpreted as experiential pathways within the specific theoretical configuration adopted in this study, rather than as evidence that the S-O-R framework can universally explain the relationships between all EC-DHA stimuli and consumer behaviors.

#### 2.2.1. Characteristics of EC-DHA (Stimulus)

Livestream e-commerce is an interactive form of electronic commerce that integrates real-time video streaming with online shopping, enabling streamers to present and promote products while responding to consumer questions in real time (H. Chen et al., 2023). Building on prior research, this study adopts a human–computer interaction perspective to conceptualize four core characteristics of e-commerce digital human avatars (EC-DHAs): interactivity, empathy, immersion, and vividness. First, interactivity is primarily reflected in real-time bidirectional feedback (Lin et al., 2025), referring to a continuous, immediate, and comprehensible interaction process between consumers and EC-DHAs. This includes timely responses to questions, clear product-related feedback, smooth interaction processes, and dynamic adjustment of recommendation content. Second, empathy is primarily reflected in emotional resonance (Gong & Sun, 2025). It refers to the extent to which EC-DHAs use affective language and approachable expressions to make consumers feel understood and acknowledged, thereby increasing their receptiveness to livestream content and product recommendations. Third, immersion refers to the attentional absorption and involvement experienced by consumers during viewing and interaction, as reflected in sustained attention to livestream content, reduced awareness of the surrounding environment, and altered perceptions of time (Jennett et al., 2008). Although immersion is related to presence and telepresence, these concepts are distinct. Presence and telepresence emphasize the spatial experience of feeling situated within a mediated environment, whereas this study focuses on consumers’ subjective involvement and absorption during EC-DHA livestreaming. Because this experience is elicited by the dynamic presentation, interactive feedback, and continuous situational flow of EC-DHA livestreams, immersion is incorporated into the Stimulus component as consumers’ subjective perception of the immersive characteristics of the livestream environment rather than as an objective environmental attribute. Finally, vividness is primarily reflected in the richness and expressiveness of visual presentation (Lin et al., 2025). It refers to the ability of EC-DHAs to present product details and usage scenarios in a concrete, clear, and expressive manner, thereby enhancing consumers’ direct perception of product information. Accordingly, this study conceptualizes EC-DHA characteristics from a human–computer interaction perspective along four dimensions: interactivity, empathy, immersion, and vividness.

#### 2.2.2. Consumer Experience (Organism)

The Organism component of the S-O-R model represents the internal cognitive, affective, and experiential states that arise in response to external environmental stimuli (Eroglu et al., 2003; Mehrabian & Russell, 1974). In the EC-DHA livestream shopping context, these internal experiences are multidimensional, encompassing evaluations of the digital human and its visual presentation, affective activation elicited by livestream interactions, and engagement developed through continued viewing and interaction. Accordingly, this study does not treat visual appeal, consumer-perceived arousal, and consumer engagement as three separate response variables. Instead, they are conceptualized as complementary dimensions of internal experience corresponding to visual evaluation, affective activation, and participatory involvement, respectively. This approach provides a context-specific refinement of the Organism component of the S-O-R model for EC-DHA livestreaming. Importantly, the Organism component in this study does not capture momentary psychological states in real time during actual livestream interactions. Rather, it is operationalized through participants’ self-reported evaluations of visual appeal, perceived arousal, and engagement after exposure to a standardized EC-DHA livestream stimulus. These constructs should therefore be interpreted as subjective experiential responses following stimulus exposure rather than as direct measures of instantaneous psychological states.

Visual appeal refers to consumers’ overall perception of attractiveness and aesthetic preference toward the visual presentation of an interface, image, or product. It is closely associated with visual complexity, order, color configuration, and presentation style (Deng & Poole, 2012; Yang et al., 2022). In live-streaming e-commerce, attractive visual design can enhance perceived visual appeal and strengthen consumers’ impulse buying tendency (Yang et al., 2022). Consumer perceived arousal is also a critical psychological indicator in live-streaming e-commerce contexts (Yang et al., 2022). In the EC-DHA context, it refers to consumers’ excitement and emotional activation triggered by EC-DHAs and live-streaming environmental stimuli (Yang et al., 2022). Such emotional arousal can reinforce unplanned purchasing tendencies during live-streaming shopping and promote impulse buying intention (M. Li et al., 2022; X. Liu et al., 2023). In addition, consumer engagement is a core component of consumer experience. It reflects consumers’ cognitive, emotional, and behavioral investment during interaction (Hollebeek et al., 2014) and significantly influences purchase intention and consumption responses (He et al., 2022).

Although these constructs are related within the EC-DHA livestreaming context, they differ in their primary conceptual focus. Interactivity reflects consumers’ perceptions of bidirectional communication, immediate feedback, and interaction control. Empathy captures consumers’ socio-emotional judgments of whether the EC-DHA understands and responds to their emotions and needs. Vividness concerns the richness, clarity, and realism of information presentation, whereas immersion reflects consumers’ sense of presence and attentional absorption within the livestream environment. By contrast, visual appeal represents an aesthetic evaluation of the EC-DHA’s appearance, interface, and visual presentation. Consumer-perceived arousal reflects the immediate psychological activation and excitement elicited by the stimulus, while consumer engagement captures cognitive, affective, and behavioral involvement during continued viewing and interaction. Thus, although these constructs may be interrelated, they represent distinct psychological and experiential dimensions, including communication processes, socio-emotional understanding, sensory presentation, immersive experience, visual evaluation, affective activation, and sustained participation. Accordingly, they possess conceptually distinct theoretical boundaries.

#### 2.2.3. Consumer Impulse Buying Intention (Response)

Consumer impulse buying intention (CIBI) refers to consumers’ sudden, immediate, and unplanned tendency to purchase in response to external marketing or situational stimuli. It reflects an impulsive purchase tendency formed through cognitive evaluation and emotional response, rather than actual purchase behavior itself (Chan et al., 2017; M. Li et al., 2022). Within the S-O-R paradigm, CIBI is regarded as a key dependent variable for assessing consumer responses. It indicates the extent to which external stimuli generate immediate and unplanned purchase intentions and reflects how consumers’ internal cognitive and emotional states are translated into potential consumption responses (C. C. Chen & Yao, 2018). Therefore, this study adopts CIBI as the core response variable, providing a consumer-behavior-based evaluation framework for EC-DHA live-streaming contexts.

Purchase intention is generally based on information comparison, rational evaluation, and planned deliberation, and refers to the likelihood that consumers will purchase a particular product or service in the future (Ajzen, 2020). By contrast, consumer impulse buying intention (CIBI) emphasizes a sudden, emotional, and unplanned tendency to purchase triggered by immediate stimuli. Consumers may initially have no clear purchase plan, but visual appeal, emotional arousal, interactive feedback, or promotional incentives can generate an immediate impulse to buy (Rook, 1987; Verplanken & Herabadi, 2001). As AI-driven digital shopping guides, EC-DHAs can rapidly influence consumers’ psychological states through real-time interaction, dynamic presentation, emotional expression, and scenario-based recommendation. Therefore, this study does not focus on purchase intention formed through sufficient rational evaluation, but rather on CIBI generated by the immediate stimulation of EC-DHAs. Compared with general purchase intention, CIBI more effectively explains consumers’ rapid, emotional, and unplanned purchase responses to emerging technological stimuli in EC-DHA live-streaming contexts.

### 2.3. Hypotheses Development and Conceptual Model Construction

#### 2.3.1. Interactivity

Interactivity refers to the extent to which consumers perceive bidirectional communication, immediate feedback, and control over participation during interactions with EC-DHAs and other users, encompassing both social interaction and human–computer interaction (Doong, 2022; Lin et al., 2025). In livestream e-commerce, interactivity is widely regarded as an important determinant of consumer experience and behavioral responses because it reflects not only the timeliness and continuity of communication but also consumers’ perceived participation and control during the interaction process (Yang et al., 2022). The concept originates from communication and human–computer interaction research on bidirectional communication and feedback mechanisms (Doong, 2022) and has been widely applied in studies of virtual product purchasing (Animesh et al., 2011), online shopping (M. J. Chen, 2023), and livestream e-commerce (Yang et al., 2022). Empirical evidence further suggests that interactivity can enhance consumer experience and satisfaction, thereby increasing purchase intention (Lin et al., 2025). In the context of e-commerce digital humans, which integrate product presentation with real-time communication, interactivity can therefore be defined as the extent to which EC-DHAs provide real-time responses, bidirectional communication, and dynamic feedback. This construct emphasizes the communication structure and feedback process and is conceptually distinct from empathy, which focuses on understanding and responding to consumers’ emotions and needs. Interactivity also differs from visual appeal, which depends primarily on the digital human’s appearance and visual presentation. Accordingly, interactivity may be more directly associated with consumer arousal and engagement, whereas its relationship with visual appeal may be comparatively weaker. Nevertheless, based on prior research, this study retains and empirically tests the proposed positive relationship. The specific hypotheses are as follows:

**H1a.**  *EC-DHA interactivity positively affects visual appeal.*

**H1b.**  *EC-DHA interactivity positively affects consumer perceived arousal.*

**H1c.**  *EC-DHA interactivity positively affects consumer engagement.*

**H1d.**  *EC-DHA interactivity positively affects consumer impulse buying intention.*

#### 2.3.2. Empathy

Empathy refers to the extent to which consumers perceive that an EC-DHA can recognize, understand, and appropriately respond to their emotions, needs, and concerns. Unlike traditional interpersonal empathy, empathy in the EC-DHA context is conveyed primarily through perceptible cues such as affective language, facial expressions, tone of voice, and adaptive responses, which can enhance consumers’ emotional and social experiences with AI avatars (Liu-Thompkins et al., 2022). Whereas interactivity concerns the timeliness and bidirectionality of communication, empathy focuses on whether consumers perceive that the EC-DHA understands and appropriately responds to their emotions, preferences, and needs. Its conceptual core therefore lies in socio-emotional understanding rather than communication frequency or response speed. Prior research highlights the importance of empathy and emotional expression in interactions between virtual avatars and consumers. Mirowska and Arsenyan (2023), for example, found that empathy influences consumers’ social media engagement with virtual influencers, while Yu et al. (2024) showed that emotional expression by AI-generated virtual influencers significantly affects user engagement. In livestream e-commerce, Bai et al. (2025) found that empathy between streamers and viewers can improve sales performance, whereas Gong and Sun (2025) reported that affective language used by virtual streamers is more effective than rational language in increasing consumers’ willingness to adopt their recommendations. H. Liu et al. (2025) further demonstrated that the interaction styles of AI-generated virtual streamers can influence purchase intention through social presence and perceived value, indicating that socio-emotional interactions with AI virtual streamers can translate into consumer responses. Overall, empathy may enhance consumers’ socio-emotional evaluations of EC-DHAs and contribute to visual evaluation, affective arousal, and engagement. However, empathy primarily reflects the socio-emotional experience of feeling understood and acknowledged and does not necessarily provide the product-related stimulation, urgency, or decision motivation required for an immediate purchase. Thus, empathy may be directly associated with impulse buying intention or may operate primarily through consumers’ internal experiential states. Based on the positive associations documented in prior research, this study proposes the following directional hypotheses while treating the existence of a direct effect independent of these experiential mechanisms as an empirical question. The specific hypotheses are as follows:

**H2a.**  *EC-DHA empathy positively affects visual appeal.*

**H2b.**  *EC-DHA empathy positively affects consumer perceived arousal.*

**H2c.**  *EC-DHA empathy positively affects consumer engagement.*

**H2d.**  *EC-DHA empathy positively affects consumer impulse buying intention.*

#### 2.3.3. Immersion

Immersion (IM) in this study refers to consumers’ perceptions of the immersive qualities of the EC-DHA and its livestream environment, specifically the extent to which dynamic presentation, real-time interaction, continuous scenes, and multimodal information make the shopping environment feel vivid, immediate, and contextually engaging. Unlike traditional static e-commerce, which relies primarily on text and images, livestream e-commerce enhances consumers’ sense of presence through dynamic presentation, real-time interaction, and continuous scene-based communication. Prior research has shown that telepresence can enhance experiential value in livestream shopping and subsequently influence consumer responses (Barta et al., 2023). M. Li et al. (2022) similarly found that social presence generated by livestream environments can promote impulse buying through consumers’ internal emotional states. Huo et al. (2023) further demonstrated that social presence enhances flow experience, which in turn promotes impulse buying behavior. In the context of virtual streamers, Gao et al. (2023) found that vividness, cuteness, and responsiveness can strengthen consumers’ telepresence and subsequently increase purchase intention. X. Li et al. (2024) also reported that streamer interaction can enhance consumer concentration and flow experience, with greater concentration increasing the likelihood of impulse buying. More recent research further suggests that immersive experience serves as an important psychological mechanism linking livestream stimuli to impulse buying responses (Y. Wang et al., 2026). Taken together, immersion in this study represents a subjective perception induced by cues in the EC-DHA livestream environment and is therefore incorporated into the Stimulus component of the operational framework. Its core focus is the extent to which dynamic presentation, interactive feedback, and continuous scenes make the livestream environment feel immediate and contextually engaging. This perceived environmental quality may subsequently be associated with consumers’ visual evaluations, affective arousal, and engagement, and ultimately with impulse buying intention. Because immersion primarily reflects consumers’ perceptions of the immediacy and contextual richness of the livestream environment, it may first influence attentional involvement, emotional experience, and engagement rather than directly translate into purchase impulses. Accordingly, immersion may be directly associated with impulse buying intention or may exert its influence primarily through visual evaluation, perceived arousal, and engagement-related experiences. The specific hypotheses are as follows:

**H3a.**  *EC-DHA immersion positively influences visual appeal.*

**H3b.**  *EC-DHA immersion positively influences consumer perceived arousal.*

**H3c.**  *EC-DHA immersion positively influences consumer engagement.*

**H3d.**  *EC-DHA immersion positively influences consumer impulse buying intention.*

#### 2.3.4. Vividness

Vividness refers to the extent to which a media environment presents information in a rich, clear, and realistic sensory manner (Steuer, 1992). In marketing research, vividness has been shown to significantly enhance consumers’ social presence, involvement, and behavioral intention (Fortin & Dholakia, 2005). It is also highly relevant in live-streaming e-commerce. Prior studies indicate that vividness strengthens consumers’ impulse buying tendency by enhancing consumption vision and presence (Khoi et al., 2023). In the EC-DHA context, improving the vividness of product presentation, scenario enactment, and digital human expression is an important strategy for enhancing consumer experience, increasing live-streaming attractiveness, and promoting impulse buying intention. The specific hypotheses are as follows:

**H4a.**  *EC-DHA vividness positively influences visual appeal.*

**H4b.**  *EC-DHA vividness positively influences consumer perceived arousal.*

**H4c.**  *EC-DHA vividness positively influences consumer engagement.*

**H4d.**  *EC-DHA vividness positively influences consumer impulse buying intention.*

#### 2.3.5. Visual Attractiveness

Visual appeal refers to consumers’ overall aesthetic evaluation and perceived attractiveness of interfaces, images, and display elements and is commonly associated with the complexity, order, and design of visual presentations (Deng & Poole, 2012). In the EC-DHA context, it specifically reflects consumers’ cognitive evaluations and affective preferences regarding visual cues such as the EC-DHA’s appearance, the livestream interface, product presentation, and the surrounding visual atmosphere (Deng & Poole, 2012; Yang et al., 2022). Visual appeal (VA) should be distinguished from vividness (VI). Vividness refers to the richness, clarity, and realism of EC-DHA information and sensory presentation, whereas visual appeal represents consumers’ subjective aesthetic evaluations and perceptions of attractiveness in response to these visual elements. Thus, vividness emphasizes presentation characteristics, while visual appeal emphasizes consumers’ evaluative responses. Unlike traditional static e-commerce webpages, livestream e-commerce involves real-time, dynamic, and multimodal visual presentations, making visual appeal particularly relevant to consumer experience and behavioral responses (Deng & Poole, 2012; Yang et al., 2022). Prior research indicates that more attractive interface designs and livestream atmospheres can enhance perceived visual appeal and subsequently influence impulse buying intention through consumer arousal and engagement (J. Chen et al., 2024). These findings provide theoretical support for examining the role of visual appeal in livestream e-commerce. The specific hypothesis is as follows:

**H5.**  *EC-DHA visual appeal positively influences consumer impulse buying intention.*

#### 2.3.6. Consumers’ Perceived Arousal

Consumer-perceived arousal refers to a heightened state of internal psychological activation elicited by EC-DHA livestream stimuli, reflected in excitement, increased attention, curiosity, and situational interest in the products presented. The construct is theoretically grounded in the arousal dimension of the PAD framework (Mehrabian & Russell, 1974) but is contextually operationalized to capture the interactive and consumption-specific characteristics of livestream e-commerce rather than directly replicating the classical PAD arousal scale. Importantly, it represents consumers’ immediate internal state following exposure to stimuli, rather than an established purchase decision or behavioral intention. Prior research has demonstrated the explanatory relevance of perceived arousal across a range of digital consumption contexts, including online store atmospherics (Eroglu et al., 2003), online impulse buying (Verhagen & Van Dolen, 2011), mobile auctions (C. C. Chen & Yao, 2018), and livestream e-commerce (Yang et al., 2022). In particular, Yang et al. (2022) showed that interface design and livestream atmosphere can influence impulse buying intention through visual appeal, perceived arousal, and consumer engagement. This mechanism is also relevant to EC-DHA contexts. When the visual presentation, interactive feedback, information delivery, and livestream atmosphere associated with EC-DHAs elicit higher levels of arousal, consumers are more likely to experience excitement, heightened involvement, and immediate responsiveness, thereby strengthening engagement and impulse buying tendencies (C. C. Chen & Yao, 2018; Yang et al., 2022). Collectively, these findings underscore the important role of consumer-perceived arousal in shaping impulse buying intention. The specific hypothesis is as follows:

**H6.**  *Consumer perceived arousal positively affects consumer impulse buying intention.*

#### 2.3.7. Consumer Engagement

Consumer engagement refers to the degree of cognitive, affective, and behavioral involvement that consumers exhibit within a specific interaction context (Hollebeek et al., 2014). As an active engagement state, it is reflected in sustained attention, emotional connection, and active participation in relation to content, brands, or livestream activities (Hollebeek et al., 2014). Although consumer engagement (CE), immersion (IM), and consumer-perceived arousal (CP) are all related to consumer experience, they represent conceptually distinct processes. IM primarily reflects consumers’ perceptions of the immersive and immediate qualities of the EC-DHA livestream environment and emphasizes the experience of being absorbed in the situation. CP refers to the immediate psychological activation elicited by stimuli, such as excitement and emotional arousal, and therefore captures the intensity of short-term affective activation. By contrast, CE reflects sustained cognitive, affective, and behavioral involvement with livestream content and interactive activities. Thus, IM emphasizes perceived immersion, CP emphasizes immediate affective activation, and CE emphasizes sustained participatory involvement. He et al. (2022) identified a significant positive relationship between consumer engagement and purchase intention in livestream e-commerce, while Zheng et al. (2022) further demonstrated that consumer engagement behaviors during livestreaming significantly influence impulse buying intention. These findings are also applicable to the EC-DHA context, in which livestream experiences that strengthen cognitive involvement, emotional connection, and interactive participation are likely to enhance consumer engagement. Collectively, prior studies highlight the important role of consumer engagement in shaping impulse buying intention. The specific hypothesis is as follows:

**H7.**  *Consumer engagement positively affects consumer impulse buying intention.*

#### 2.3.8. Conceptual Model

**Figure 2:**
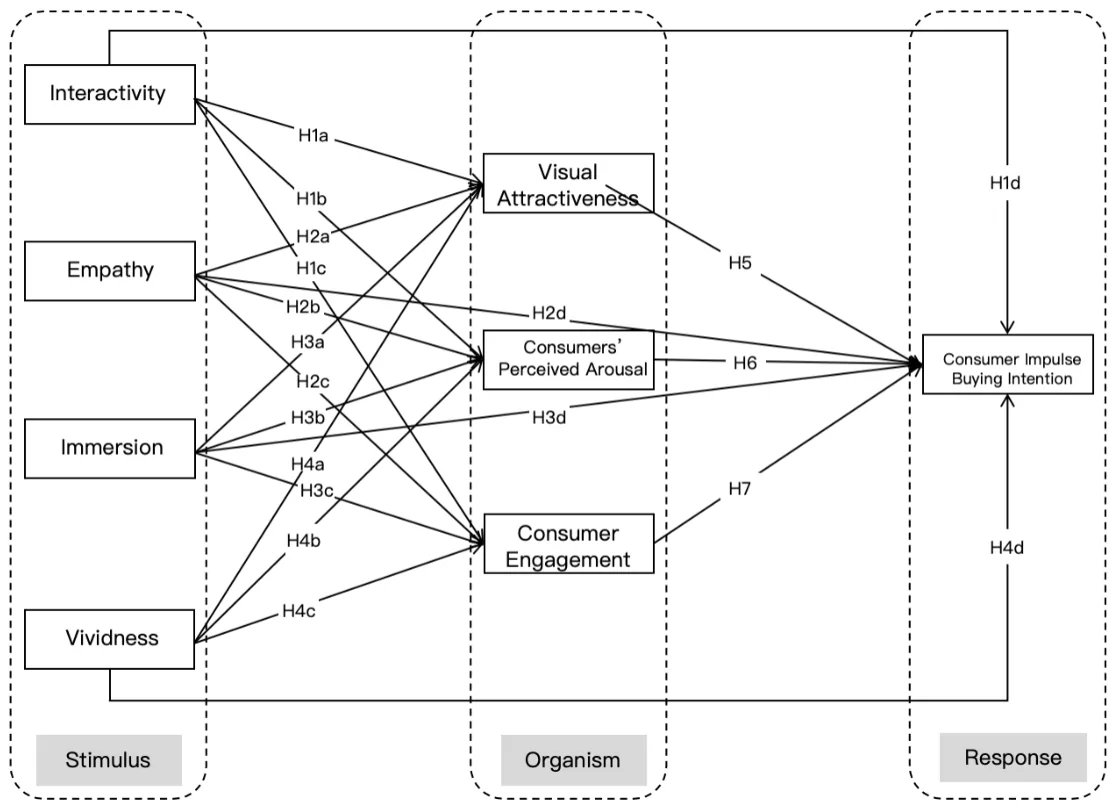
Hypothesized conceptual model.

Based on the S-O-R framework, this study examines how distinct stimulus factors (S: interactivity, empathy, immersion, and vividness) influence organismic states (O: visual appeal, consumer-perceived arousal, and consumer engagement), which in turn are associated with the response (R: consumer impulse buying intention in the EC-DHA context). Importantly, interactivity, empathy, immersion, and vividness are not treated as indicators of a single higher-order construct of “EC-DHA characteristics.” Instead, they are conceptualized as four theoretically distinct stimulus dimensions; therefore, high correlations among them are neither assumed nor required. Similarly, the structural model does not assume that all EC-DHA characteristics operate through identical mechanisms. Rather, the specified paths are intended to compare how different stimuli are associated with consumer responses through distinct experiential pathways. Figure 2 presents the proposed conceptual model.

## 3. Methodology

### 3.1. Research Design

**Figure 3:**
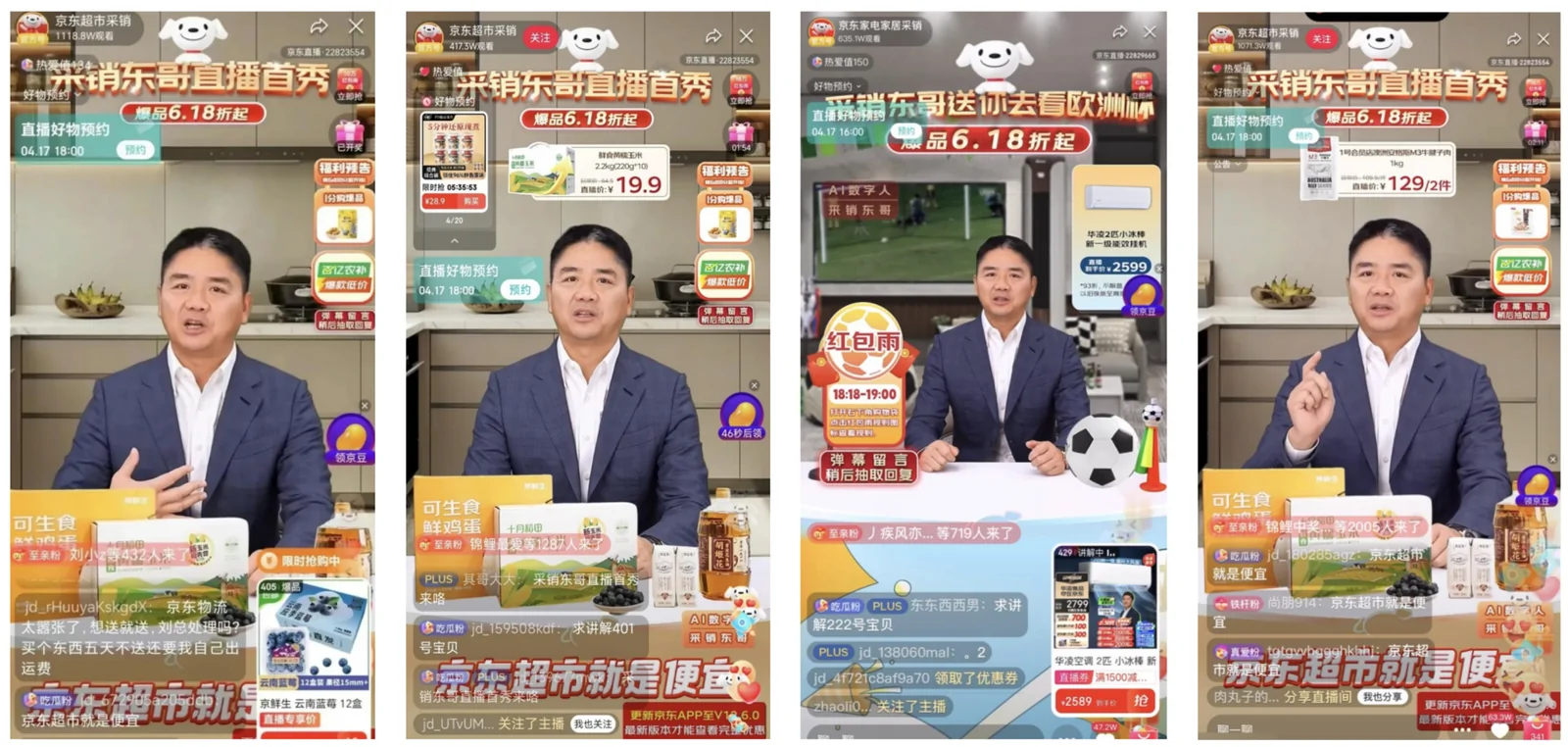
JD Supermarket AI Digital Human Livestream Featuring “Caixiao Dongge”. Note: “Caixiao Dongge” is the designated name of JD Supermarket’s AI digital human streamer. The Chinese text visible in the screenshots consists of original livestream interface elements, such as promotional information, product information, and platform labels. These elements are retained to preserve the original livestream context and are not used as analytical content in this study. Source: Livestream screenshots from JD Supermarket’s “Caixiao Dongge” AI digital human livestream.

To investigate how EC-DHAs shape consumers’ cognitive evaluations, affective responses, and impulse buying intention in livestream shopping, this study uses Liu Qiangdong’s AI digital human livestream on JD.com as the research context (see Figure 3). The livestream is modeled on the company founder and employs AI digital human technology to simulate the appearance, speaking style, and interactive behaviors of a human streamer. It creates an immersive shopping environment through product presentation, promotional information, real-time interaction, and purchase guidance. This case provides a typical livestream e-commerce setting, demonstrates salient applications of AI digital human technology, and has attracted substantial consumer attention. It is therefore adopted as a specific EC-DHA livestream context for examining consumers’ perceptions of the relationships among EC-DHA characteristics, consumer experience, and impulse buying intention. However, the case is primarily intended to provide a relatively consistent research setting and should not be regarded as representative of all e-commerce platforms, EC-DHA types, or livestreaming formats. Moreover, “Caixiao Dongge” is an EC-DHA modeled on a real corporate founder and therefore differs from generic, hyper-realistic, or anime-style digital humans. Consumer evaluations of this EC-DHA may also be influenced by factors such as brand reputation and familiarity with the individual, whereas evaluations of generic or anime-style digital humans may depend more strongly on visual design, character configuration, and interactive performance.

Methodologically, this study adopts a sequential explanatory mixed-methods design. The theoretical model and relationships among variables are first tested through quantitative analysis, followed by qualitative inquiry to interpret and supplement the quantitative findings. The research design consists of two interconnected sub-studies. Study 1 uses a questionnaire survey to test the path relationships among variables in the EC-DHA live-streaming context and identify the key factors influencing consumers’ impulse buying intention. Study 2 uses semi-structured interviews to further explain the results of Study 1 and reveal consumers’ perceived experiences, participatory responses, and impulse buying motivations when watching EC-DHA live streams. This design not only identifies relationships among variables but also deepens understanding of the mechanism through which EC-DHA live streaming influences consumers’ impulse buying intention, consistent with the logic of explanation-oriented mixed-methods research (Venkatesh et al., 2013).

In the quantitative phase, this study adopts an analytical framework that integrates partial least squares structural equation modeling (PLS-SEM) and artificial neural networks (ANN). First, PLS-SEM is used to test the structural relationships among latent variables and evaluate the research hypotheses. This approach reveals the path relationships among EC-DHA live-streaming experience, consumers’ psychological evaluations, and impulse buying intention at the overall model level. Second, ANN is employed to identify potential nonlinear predictive patterns and enhance the model’ s predictive capability for key outcome variables. By combining these two methods, this study explains the mechanisms among variables from a structural perspective and identifies key factors influencing consumers’ perceived experience, participatory responses, and impulse buying intention from a predictive perspective. This integrated approach improves the explanatory depth and robustness of the findings.

**Figure 4:**
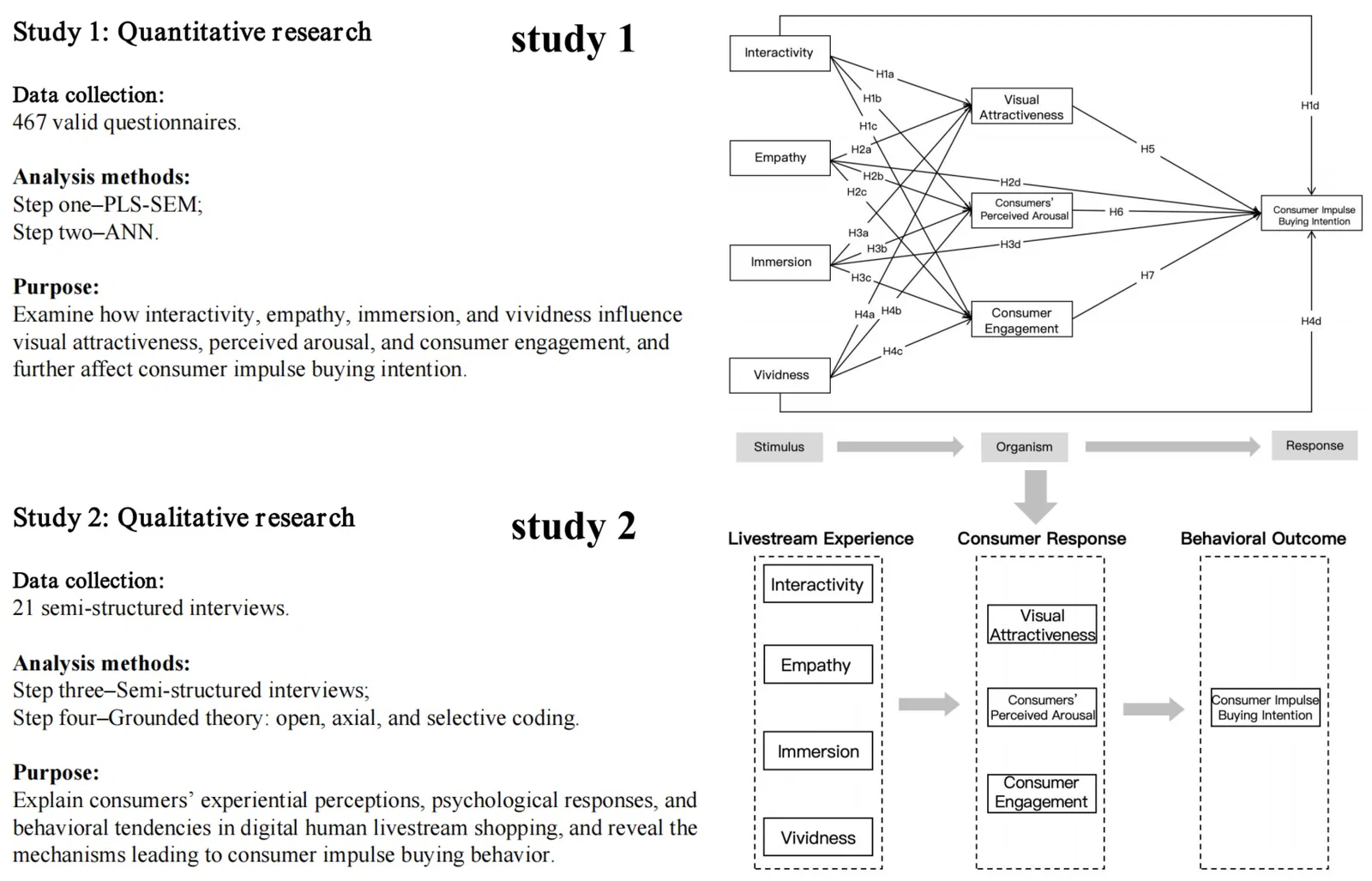
Research model with explanatory sequential mixed-methods design. Source: Authors’ own compilation.

In the qualitative phase, follow-up semi-structured interviews were conducted based on the findings of Study 1, and the interview data were analyzed using grounded theory-informed coding procedures. This phase focused on consumers’ perceived experiences, emotional responses, and interpretations of impulse buying intention during EC-DHA livestream viewing. Its primary aim was to provide a more context-sensitive explanation of the key relationships identified in the quantitative analysis and to uncover potential mechanisms not fully captured by the original structural model. By integrating the quantitative and qualitative components within a single research framework, the two phases complemented each other and strengthened the study’s explanation of how EC-DHA livestreaming is associated with consumers’ impulse buying intention. The overall research design is presented in Figure 4.

### 3.2. Study 1: Quantitative Phase

#### 3.2.1. Instrumentation

The questionnaire was developed based on established theoretical models and validated measurement scales. All items were adapted from previously validated instruments and moderately revised to fit the EC-DHA livestream shopping context, thereby enhancing contextual relevance. Table 2 summarizes the conceptual definitions, number of items, adapted measurement content, and corresponding references for each construct. All items were measured on a seven-point Likert scale ranging from 1 = Strongly Disagree to 7 = Strongly Agree. Specifically, the Livestream Experience constructs (IN, EM, IM, and VI) were adapted from Y. Liu (2003), Davis (1983), Jennett et al. (2008), and Steuer (1992), respectively. The Consumer Response constructs (VA, CP, and CE) were adapted from Cyr et al. (2006), Mehrabian and Russell (1974), and Dessart et al. (2015), respectively, while the Behavioral Outcome construct (CIBI) was adapted from Rook (1987). Because the original scales were developed in English and the survey was administered in Chinese, the original items were first translated into Chinese and then contextually reworded for EC-DHA livestream shopping. The translated and adapted items were subsequently reviewed by five individuals with relevant academic backgrounds, including two graduate students in related fields, two doctoral researchers in design, and one design professor. The review focused on semantic clarity, construct alignment, and contextual suitability. Based on their feedback, several items were refined while preserving the core theoretical meaning of each construct. The Immersion (IM) scale was adapted from Jennett et al. (2008) and primarily assesses attentional absorption, involvement, and altered time perception during EC-DHA livestreaming. Accordingly, IM is conceptually distinct from spatial presence and telepresence, which primarily emphasize the spatial sense of being present within a mediated environment. In this study, IM specifically reflects consumers’ immersive involvement in the EC-DHA livestream interaction process.

Consumer engagement (CE) was measured using three items, whereas most other constructs were measured using four. The number of items for each construct was determined by the original measurement scales and their suitability for the EC-DHA livestreaming context, rather than standardized artificially across constructs. Because the number of items can affect Cronbach’s α and thereby limit direct comparisons across constructs, the measurement quality of CE was assessed using multiple indicators, including composite reliability (CR), average variance extracted (AVE), and factor loadings.

Further clarification is warranted regarding consumer-perceived arousal (CP), consumer engagement (CE), and consumer impulse buying intention (CIBI). Although these constructs are related, they represent distinct theoretical dimensions. CP captures the immediate psychological activation elicited by EC-DHA livestream stimuli, including excitement, heightened attention, curiosity, and situational product interest. In particular, the “purchase interest” reflected in CP1 denotes stimulus-induced product attention and psychological activation rather than a formed purchase decision or explicit purchase intention. CE, by contrast, reflects the cognitive, affective, and participatory involvement that develops during viewing and interaction, including information acquisition, sustained attention, and interactive engagement. CIBI, in turn, refers to consumers’ behavioral intention to make an immediate, impulsive, or unplanned purchase. Accordingly, CP and CE represent internal experiential states within the Organism component of the S-O-R framework, whereas CIBI represents a behavioral response tendency within the Response component. These constructs are therefore conceptually distinct despite their potential interrelationships.

#### 3.2.2. Data Collection

This study employed a purposive sampling survey to empirically test the proposed research model. The core constructs examined, including interactivity, empathy, immersion, and vividness, reflect consumers’ subjective perceptions and psychological responses during EC-DHA livestream shopping. Because these constructs are latent and cannot be directly observed, they were measured using structured self-report questionnaires, a commonly used approach for assessing psychological constructs (Spector, 1994). Moreover, existing public databases do not provide systematic data on consumers’ psychological responses, engagement experiences, or impulse buying intentions in EC-DHA livestreaming contexts, making primary data collection through a questionnaire survey necessary. All research procedures were conducted in accordance with relevant guidelines and regulations. Before participation, all participants were clearly informed of the study purpose and their rights. Ethical approval was obtained from the Institutional Review Board of Hanyang University before formal data collection, and written informed consent was obtained from all participants to ensure the protection of their privacy.

During the pilot-testing stage, a pretest was conducted in April 2026 to assess the contextual suitability and reliability of the measurement instrument. Twenty-eight participants with prior experience watching EC-DHA livestreams were recruited through random sampling, and the collected data were subjected to reliability analysis. The results showed that Cronbach’s α and composite reliability (CR) values for all latent constructs exceeded the recommended threshold of 0.70, indicating satisfactory internal consistency and supporting the use of the instrument in the subsequent formal survey. The 28 pretest participants were included solely for scale evaluation and were excluded from the formal survey. All 467 valid responses in the formal survey were obtained from an independent sample; therefore, there was no overlap between the two samples.

The formal survey was conducted from April to May 2026. Chinese consumers were selected as the target population because EC-DHA livestreaming has been widely adopted on Chinese e-commerce platforms, where consumers have substantial exposure to livestream shopping and impulse buying contexts. Data were collected through both online and offline channels. The online questionnaire was distributed via the Wenjuanxing platform, while paper-based questionnaires were used to supplement offline data collection. To assess the comparability of the two data sources, the online and offline samples were compared in terms of demographic characteristics and key study variables. No significant differences were observed between the two groups (*p* > 0.05), supporting their combination for subsequent analyses.

Given the use of purposive sampling, the sampling strategy was designed to ensure that participants had sufficient experience to understand the EC-DHA livestreaming context rather than to generate a probability-based sample representative of the broader Chinese consumer population. Accordingly, the findings are primarily applicable to consumers with prior experience of EC-DHAs or livestream shopping and should not be generalized directly to all Chinese consumers. Although this approach improves the fit between the sample and the research context, it also limits the statistical generalizability of the findings. Potential nonresponse bias can be further assessed by comparing early and late respondents across the key study variables. If no significant differences are observed, the potential influence of nonresponse bias on the findings can be considered limited.

**Figure 5:**
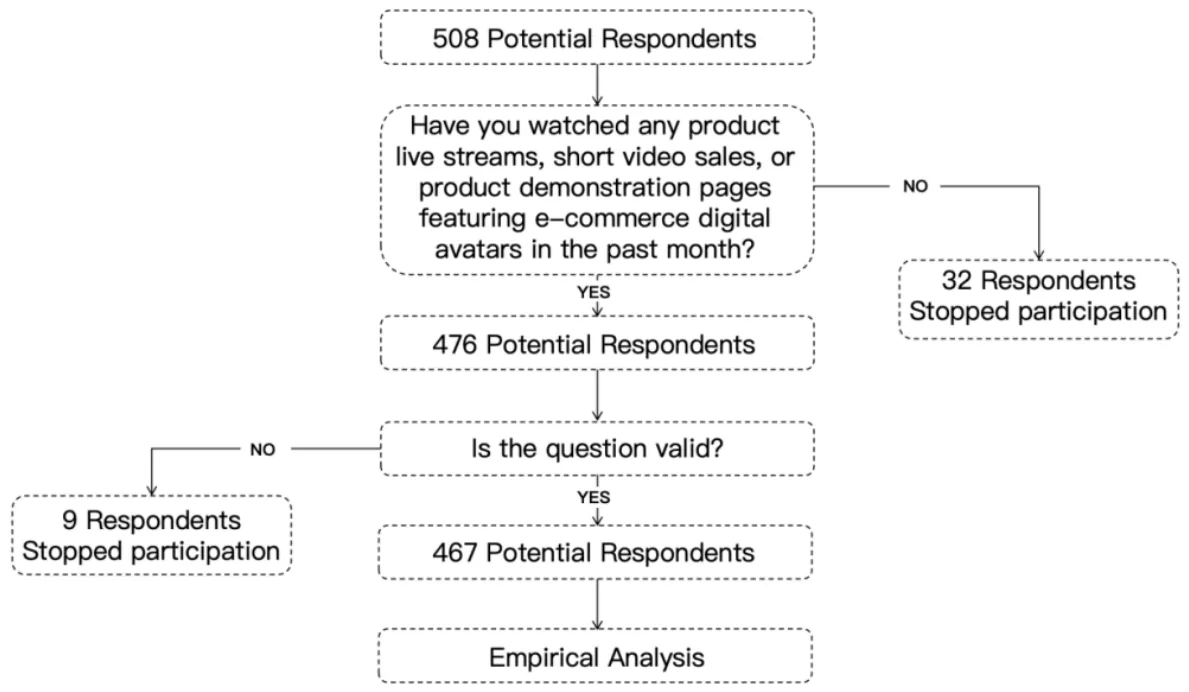
Data collection procedure. Source: Authors’ own compilation.

To minimize differences in participants’ understanding of the research context, all participants viewed the same standardized “Caixiao Dongge AI Digital Human” livestream video before completing the formal questionnaire, thereby establishing a consistent EC-DHA livestreaming context. Using a standardized stimulus helps reduce potential confounding effects arising from variations in livestream content. However, the video cannot fully replicate real-time question-and-answer exchanges, dynamic responses, or social interactions among consumers in an actual livestream. Accordingly, this study assesses participants’ self-reported subjective experiences after exposure to a standardized EC-DHA stimulus rather than momentary psychological states measured during real-time interaction. In particular, visual appeal, consumer-perceived arousal, and consumer engagement were assessed through post-exposure self-reports and may therefore be influenced by memory reconstruction, situational imagination, and common method effects. Given this measurement approach, the Organism component should be interpreted as representing perceived experiential responses following stimulus exposure rather than real-time psychological states during an actual livestream. Participants were required to meet two criteria: (1) having at least one prior experience of watching an EC-DHA livestream and (2) being able to complete the questionnaire independently and in full. The questionnaire included informed consent and screening items, demographic information, and measurement items for eight latent constructs (IN, EM, IM, VI, VA, CP, CE, and CIBI). Figure 5 illustrates the overall sample screening and data collection procedure.

#### 3.2.3. Respondent Profile

The minimum required sample size was estimated using G*Power version 3.1.9.7 based on statistical power analysis (Faul et al., 2009). based on statistical power analysis (Faul et al., 2009). With an effect size of 0.15, seven predictors, and a target statistical power of 0.95, the results indicated that at least 153 participants were required. Following Guenther et al.’s (2023) recommendations for applying and reporting PLS-SEM, sample size was evaluated in relation to model complexity, the number of path relationships, and the requirements of the statistical analysis. Initially, 508 individuals agreed to participate in this study. Before completing the questionnaire, all participants were informed that the survey was anonymous and voluntary and that they could withdraw at any stage without penalty. After informed consent was obtained, 32 participants were excluded because they had never watched or experienced EC-DHA live-streaming shopping. Consequently, 476 respondents met the eligibility criteria and completed the questionnaire. After incomplete and abnormal responses were removed, 467 valid questionnaires remained, satisfying the sample size requirement for the subsequent PLS-SEM analysis.

In terms of demographic characteristics, 45.40% of respondents were male and 54.60% were female. The largest age group was 18–25 years, representing 37.90% of the sample. Respondents with a bachelor’s degree constituted the largest educational group (49.46%). Participants also represented diverse occupational backgrounds, indicating a relatively heterogeneous sample composition. Detailed demographic characteristics are reported in Table A1.

#### 3.2.4. Data Analysis Tools

The integrated PLS-SEM and ANN approach adopted in this study is explained in the following sequential manner:

PLS-SEM: In this study, PLS-SEM was used to examine the structural relationships among consumers’ perceived experiences, psychological responses, and impulse buying intention in the EC-DHA live-streaming context. Because the research model includes multiple latent constructs and focuses on hypothesis testing and predictive assessment, PLS-SEM is appropriate for the analytical objectives of this study. Following Guenther et al.’s (2023) recommendations for applying and reporting PLS-SEM, SmartPLS 4.1 was used to evaluate the model. A two-stage analytical procedure was adopted to assess the measurement model and structural model separately. The measurement model assessment examined the reliability, convergent validity, and discriminant validity of the latent constructs to ensure that the measurement items adequately reflected the research constructs. Specifically, composite reliability (CR) and Cronbach’ s alpha were used to assess internal consistency, average variance extracted (AVE) was used to evaluate convergent validity, and the HTMT ratio and variance inflation factor (VIF) were used to assess discriminant validity and potential multicollinearity. The structural model assessment used path coefficients and the predictive relevance indicator ($Q^{2}$) to analyze the relationships among latent variables and further evaluate the model’ s explanatory power and predictive capability.

ANN: Although structural equation modeling is effective for explaining multivariate linear relationships among variables, its predictive capability is limited when modeling non-compensatory and nonlinear relationships. It may therefore oversimplify consumers’ complex decision-making processes in EC-DHA live-streaming shopping (Aghaei et al., 2023). To address this limitation, this study uses artificial neural networks (ANN) as a complementary analytical method to enhance nonlinear modeling and improve the prediction of consumers’ impulse buying intention (Aghaei et al., 2023; Sohaib et al., 2019). Specifically, the significant independent variables identified through PLS-SEM were used as input neurons in the ANN model, while the dependent variable was set as the output neuron. The ANN analysis was implemented using the multilayer perceptron (MLP) model in the SPSS Neural Networks module. During model training, the dataset was divided into training and testing sets at a ratio of 9:1, and ten-fold cross-validation was applied to reduce the risk of overfitting (Hidayat-ur-Rehman et al., 2022). Model performance was evaluated using root mean square error (RMSE). In addition, sensitivity analysis was conducted to calculate the normalized relative importance of each predictor, thereby identifying the key factors influencing consumers’ impulse buying intention. In recent years, the hybrid SEM–ANN approach has been widely applied in research on complex technology acceptance, AI applications, and consumer behavior because it enables both linear explanation and nonlinear prediction (Soomro et al., 2025).

Finally, this study integrates and interprets the PLS-SEM and ANN results to examine the research model and variable relationships from complementary analytical perspectives. This approach combines structural explanation with predictive capability. The hybrid analytical framework deepens the explanation of relationships among consumers’ perceived experiences, psychological responses, and impulse buying intention, while enhancing the model’ s predictive applicability in the complex consumption context of EC-DHA live streaming. Prior studies have confirmed the feasibility and methodological advantages of the SEM–ANN hybrid approach in research on technology adoption, mobile payment, educational satisfaction, and broader methodological applications. These studies suggest that the hybrid approach can help address the trade-off between explanatory power and predictive accuracy often associated with single-method designs (Aghaei et al., 2023; Albahri et al., 2022; Hidayat-ur-Rehman et al., 2022; Sohaib et al., 2019). However, limited research has applied an integrated PLS-SEM and ANN framework to examine consumers’ impulse buying intention in EC-DHA live-streaming contexts. Accordingly, this study offers methodological value by integrating and applying these two methods to a new research context. It provides a replicable analytical framework for explaining the formation of consumers’ perceived experiences, psychological responses, and impulse buying intention in EC-DHA live streaming, and offers methodological guidance for future empirical research in related fields.

### 3.3. Study 2: Qualitative Phase

To deepen the interpretation of the quantitative findings and further elucidate the psychological responses and behavioral tendencies that emerge during EC-DHA livestream shopping, Study 2 employed a grounded theory-informed qualitative analysis building on the results of Study 1. Semi-structured interviews were conducted, and consumers’ experiential perceptions, emotional responses, and impulse buying tendencies were systematically analyzed using coding procedures informed by grounded theory, including open, axial, and selective coding. Importantly, the purpose of this qualitative phase was not to generate a new grounded theory, but to provide a contextualized interpretation of the theoretically specified structural model examined in Study 1 while remaining open to complementary themes not captured by the quantitative model.

#### 3.3.1. Procedure

**Figure 6:**
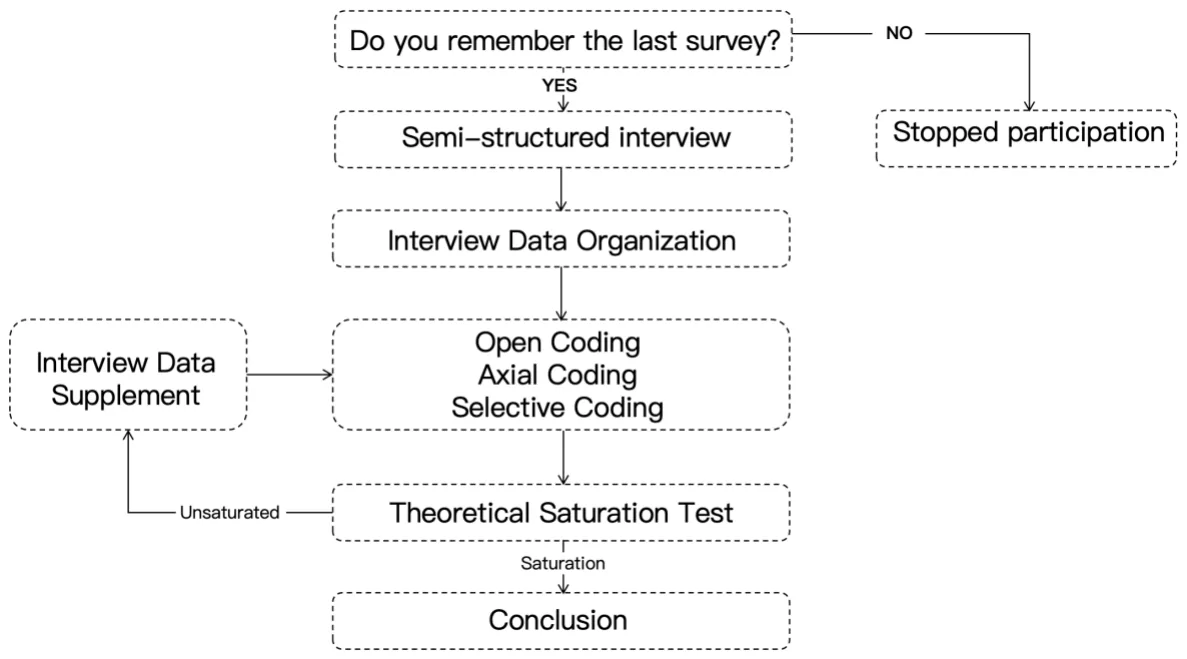
Grounded theory-informed coding procedure. Source: Authors’ own compilation.

To minimize recall bias, this study used a preliminary question (R1) before the formal interview to confirm whether respondents remembered the questionnaire survey they had previously completed. Follow-up interviews were conducted only with respondents who provided an affirmative response. Interview invitations were sent via WeChat, along with an invitation letter, a research information sheet, and an informed consent form. These materials included the research purpose, ethics approval information, confidentiality and anonymity statements, and recording permission details. After obtaining respondents’ consent, the research team confirmed the specific interview time with each participant. Given participants’ different geographical locations, all interviews were conducted online in a semi-structured format using Zoom or Tencent Meeting. Each interview lasted approximately 15–20 min. To ensure the relevance and validity of the interview data, all respondents were required to meet three criteria: they had to meet the basic screening criteria of Study 1, participate in the full interview process, and clearly express and recall their own experiences. With respondents’ permission, all interviews were audio-recorded and subsequently transcribed verbatim for analysis. The grounded theory analytical procedure is shown in Figure 6.

#### 3.3.2. Sample

Study 2 employed a combination of purposive and theoretical sampling. Based on the screening criteria established in Study 1, follow-up interviews were conducted with a subset of respondents who had previously completed the questionnaire survey. A total of 25 potential interviewees were contacted, of whom four were excluded because they had only limited recollection of the earlier questionnaire content. The final qualitative sample therefore comprised 21 participants. The sample included individuals from diverse occupational backgrounds, including students, teachers, and designers, thereby providing a degree of cross-disciplinary diversity. Participants were primarily active on major social media platforms such as Xiaohongshu, Douyin, Weibo, Bilibili, and WeChat, reflecting varied digital-use contexts and cultural experiences. Participant characteristics are summarized in Table A2. Notably, the three participants currently residing in South Korea were all Chinese consumers. The regional information reported in Table A2 refers to participants’ current place of residence rather than nationality; therefore, these participants remained consistent with the study’s sampling criteria.

This study retained variation in participants’ age and occupational backgrounds to capture a broader range of perspectives on EC-DHA livestream shopping experiences. The heterogeneous sample was intended to broaden the range of consumer experiences represented in the qualitative data rather than to support demographic group comparisons or moderation analyses. Accordingly, the study does not infer individual-difference effects from interview responses across age or occupational groups.

#### 3.3.3. Semi-Structured Interview Questions

The semi-structured interview protocol was systematically developed based on the research model and relevant literature. First, “Digital Human Avatar” and “Impulse Buying Intention” were used as core keywords to search the Web of Science database. Representative studies were then selected according to citation frequency and download counts. VOSviewer was subsequently used to conduct bibliometric analysis and identify key topics and core concepts in the field. Based on this process, the interview questions were designed to guide respondents in elaborating on their perceived experiences, emotional responses, and impulse buying intention in EC-DHA live-streaming shopping contexts. Specifically, the interview covered respondents’ overall perceptions of EC-DHA live-streaming experiences, their understanding of digital human streamers’ interaction styles and product presentation methods, and the influence of these experiences on perceived visual appeal, emotional arousal, and consumer engagement. It also examined whether these experiences further affected respondents’ impulse buying intention and actual purchase intention. Open-ended questions were used throughout the interviews, with appropriate follow-up questions asked according to respondents’ answers. This approach maintained consistency in the interview structure while capturing individualized consumption experiences and their potential underlying mechanisms.

## 4. Study 1. Quantitative Study Results

### 4.1. Common Method Bias

Common method bias is a systematic, non-substantive source of variance that may distort observed relationships among study variables. It can arise from questionnaire design, single-source data collection, respondent tendencies, and measurement context, thereby threatening the validity of survey-based empirical findings (Podsakoff et al., 2012; Spector, 2006). To assess this potential concern, Harman’s single-factor test was conducted using SPSS 27.0 (Fuller et al., 2016). The results identified eight factors with eigenvalues greater than 1, collectively explaining 75.190% of the total variance. The first factor accounted for 19.262% of the variance, well below the 40% threshold. These results suggest that severe common method bias is unlikely to represent a major concern in the present study (Aguirre-Urreta & Hu, 2019).

### 4.2. PLS-SEM Results

#### 4.2.1. Assessment of Measurement Model

First, both composite reliability (CR) and Cronbach’s α exceeded the recommended threshold of 0.70, indicating satisfactory internal consistency across all latent constructs (Bagozzi & Yi, 1988; Hair et al., 2019) (see Table 3). Although CE was measured using only three items, its Cronbach’s α (0.816), CR (0.826), and AVE (0.730) all met the recommended criteria, and all item loadings exceeded 0.80, supporting satisfactory internal consistency and convergent validity. Second, the multicollinearity assessment showed that all variance inflation factor (VIF) values were below 5, indicating no serious multicollinearity among the variables (Hair et al., 2019; O’Brien, 2007). Regarding convergent validity, all indicator loadings exceeded 0.70 and all average variance extracted (AVE) values were above 0.50. Discriminant validity was further assessed using the relevant construct-level criteria reported in the subsequent analyses (Henseler et al., 2009).

Discriminant validity was assessed using both the Fornell–Larcker criterion (Table 4) and the heterotrait–monotrait ratio (HTMT) (Table 5). According to the Fornell–Larcker criterion, the square root of the average variance extracted (AVE) for each latent construct exceeded its correlations with all other constructs, indicating satisfactory discriminant validity (Hair et al., 2019; Voorhees et al., 2016). The HTMT analysis further showed that all values were below the recommended threshold of 0.90, providing additional evidence for adequate discriminant validity (Henseler et al., 2015). These findings indicate that although interactivity, empathy, immersion, vividness, visual appeal, consumer-perceived arousal, and consumer engagement are theoretically related, they remain empirically distinct at the measurement level, consistent with the conceptual boundaries established earlier. In particular, the HTMT values for CE–CIBI and CP–CIBI were 0.249 and 0.269, respectively, both well below the recommended threshold of 0.90. These results further confirm that consumer engagement and consumer-perceived arousal are empirically distinguishable from consumer impulse buying intention.

Notably, Table 4 shows that several stimulus constructs exhibit low or near-zero correlations. Interactivity, empathy, immersion, and vividness are not conceptualized as indicators of a single higher-order EC-DHA construct; rather, they are treated as theoretically distinct stimulus dimensions. Accordingly, high intercorrelations among these constructs are neither assumed nor required. Their relatively low correlations therefore provide additional empirical support for the distinctiveness of the individual stimulus dimensions.

#### 4.2.2. Assessment of Structural Model

To further assess potential collinearity arising from the multiple-predictor structure, the inner variance inflation factor (VIF) values of the predictor constructs were examined. All inner VIF values were below the recommended threshold, indicating no serious multicollinearity among the predictors in the structural model. The model’s explanatory power and predictive relevance were assessed using the coefficient of determination ($R^{2}$) and Stone–Geisser’s $Q^{2}$, respectively. $R^{2}$ represents the proportion of variance in each endogenous construct explained by its predictors, whereas $Q^{2}$ values greater than zero indicate predictive relevance for the corresponding endogenous constructs (Hair et al., 2019; Shmueli et al., 2019).

As shown in Table 6, consumer impulse buying intention (CIBI) had the highest $R^{2}$ value (0.219). Within the Organism component, the $R^{2}$ values for consumer-perceived arousal (CP), visual appeal (VA), and consumer engagement (CE) were 0.161, 0.105, and 0.094, respectively. These relatively low values indicate that the EC-DHA characteristics included in the model explain only a limited proportion of the variance in consumers’ internal experiential states. Overall, the $R^{2}$ values suggest that the model has modest explanatory power, accounting for only part of the variance in the endogenous constructs and implying that other relevant factors may not be captured by the current model. Nevertheless, all $Q^{2}$ values were greater than zero, indicating predictive relevance for each endogenous construct. CP exhibited the highest $Q^{2}$ value (0.143), followed by CIBI (0.129), VA (0.090), and CE (0.075). Accordingly, the findings should primarily be interpreted as identifying patterns of association among EC-DHA characteristics, consumer experience, and impulse buying intention rather than as evidence of strong overall explanatory power.

**Figure 7:**
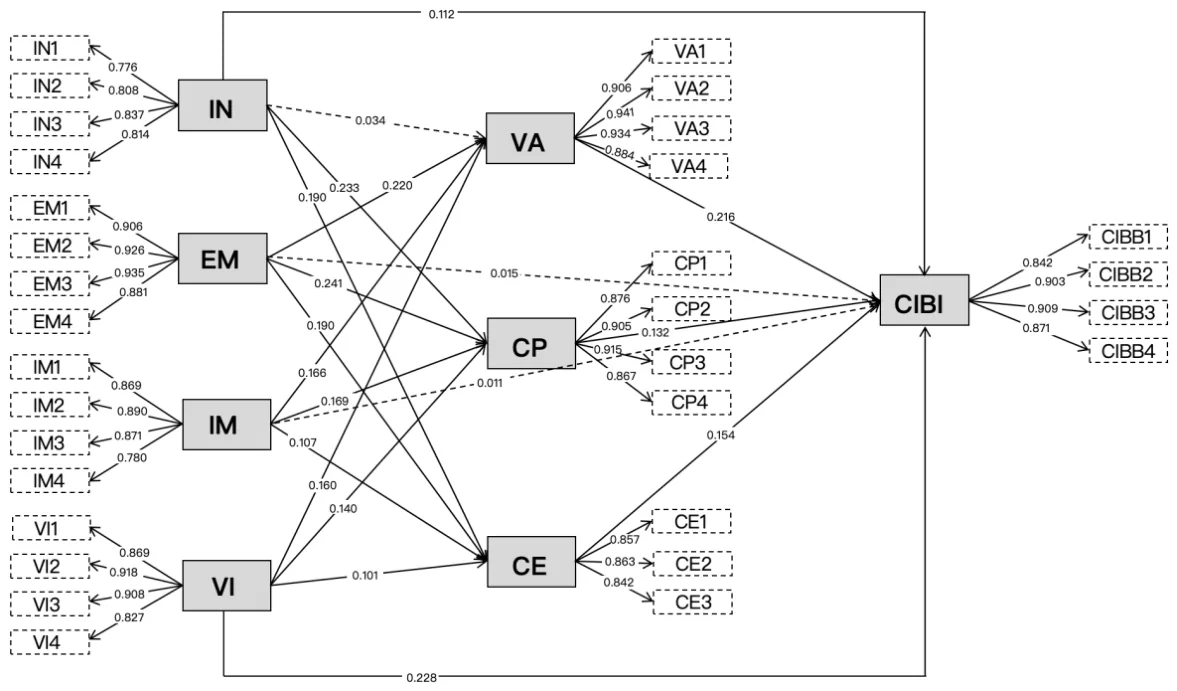
PLS results of the structural model.

As shown in Table 7, 16 hypotheses were supported. At the EC-DHA characteristic level, Empathy and Immersion were significantly and positively associated with Visual Attractiveness, Consumers’ Perceived Arousal, and Consumer Engagement (*p* < 0.05). Interactivity was significantly and positively associated with Consumers’ Perceived Arousal, Consumer Engagement, and Consumers’ Impulse Buying Intention (*p* < 0.05), whereas Vividness was significantly and positively associated with Visual Attractiveness, Consumers’ Perceived Arousal, Consumer Engagement, and Consumers’ Impulse Buying Intention (*p* < 0.05). At the consumer experiential level, Visual Attractiveness, Consumers’ Perceived Arousal, and Consumer Engagement were all significantly and positively associated with Consumers’ Impulse Buying Intention (*p* < 0.05). As illustrated in Figure 7, the model explained 9.4% of the variance in Consumer Engagement ($R^{2}$ = 0.094), 16.1% in Consumers’ Perceived Arousal ($R^{2}$ = 0.161), 10.5% in Visual Attractiveness ($R^{2}$ = 0.105), and 21.9% in Consumers’ Impulse Buying Intention ($R^{2}$ = 0.219).

Statistical significance should not be equated with substantive importance. Some significant paths exhibited relatively small standardized coefficients, such as IN→CIBI (β = 0.112) and VI→CE (β = 0.101). These results should therefore be interpreted as statistically significant but modest in magnitude, rather than as evidence of strong practical effects.

#### 4.2.3. Mediating Effect Test

To further examine the mechanisms through which EC-DHA characteristics influence consumers’ impulse buying intention, this study used bootstrapping to test the model’ s specific indirect effects. The results are presented in Table 8.

The results show that empathy had a significant indirect effect on consumer impulse buying intention through visual appeal (EM→VA→CIBI: β = 0.047, T = 3.455, *p* = 0.001), indicating that visual appeal significantly mediated the relationship between empathy and consumer impulse buying intention. Immersion and vividness also influenced consumer impulse buying intention through visual appeal. Specifically, the indirect effects of immersion (IM→VA→CIBI: β = 0.036, T = 2.874, *p* = 0.004) and vividness (VI→VA→CIBI: β = 0.035, T = 2.799, *p* = 0.005) were significant. By contrast, the indirect effect of interactivity on consumer impulse buying intention through visual appeal was not significant (IN→VA→CIBI: β = 0.007, T = 0.751, *p* = 0.453). EC-DHA characteristics also exerted significant indirect effects on consumer impulse buying intention through consumer perceived arousal. Empathy had a significant indirect effect through consumer perceived arousal (EM→CP→CIBI: β = 0.032, T = 2.489, *p* = 0.013), as did interactivity (IN→CP→CIBI: β = 0.031, T = 2.440, *p* = 0.015). Immersion and vividness also significantly influenced consumer impulse buying intention through consumer perceived arousal, with significant effects for IM→CP→CIBI (β = 0.022, T = 2.241, *p* = 0.025) and VI→CP→CIBI (β = 0.018, T = 2.023, *p* = 0.043). These findings indicate that consumer perceived arousal is an important mediator linking EC-DHA characteristics to consumer impulse buying intention. Regarding the consumer engagement pathway, empathy and interactivity had significant indirect effects on consumer impulse buying intention through consumer engagement. Specifically, the indirect effect of empathy through consumer engagement was significant (EM→CE→CIBI: β = 0.029, T = 2.453, *p* = 0.014), as was the indirect effect of interactivity (IN→CE→CIBI: β = 0.029, T = 2.491, *p* = 0.013). By contrast, the indirect effects of immersion and vividness through consumer engagement were not significant, including IM→CE→CIBI (β = 0.016, T = 1.925, *p* = 0.054) and VI→CE→CIBI (β = 0.016, T = 1.829, *p* = 0.067). Overall, the effects of EC-DHA characteristics on consumer impulse buying intention are not entirely direct. Rather, they operate through experiential pathways involving visual appeal, consumer perceived arousal, and consumer engagement. Among these pathways, visual appeal and consumer perceived arousal showed more stable mediating effects, whereas the mediating role of consumer engagement was mainly reflected in the empathy and interactivity pathways.

As shown in Table 9, the total indirect effects of EM, IM, IN, and VI on CIBI were 0.108, 0.074, 0.067, and 0.069, respectively, with corresponding VAF values of 87.10%, 86.05%, 37.43%, and 23.31%. For EM and IM, the direct effects on CIBI were nonsignificant, whereas the total indirect effects were significant. Their high VAF values indicate that, within the specified model, the relationships of EM and IM with CIBI were primarily mediated through consumer experience variables. However, these VAF values represent the relative contribution of the specified mediation pathways and should not be interpreted as evidence that the experiential mechanisms proposed within the S-O-R framework fully explain the effects of EM and IM on impulse buying intention. By contrast, both the direct and total indirect effects of IN and VI on CIBI were significant, with VAF values of 37.43% and 23.31%, respectively, indicating partial mediation. Overall, empathy and immersion were associated with impulse buying intention primarily through indirect experiential pathways, whereas interactivity and vividness were associated with impulse buying intention through both direct and mediated pathways.

### 4.3. ANN Results

#### 4.3.1. Model Specification and Development

**Figure 8:**
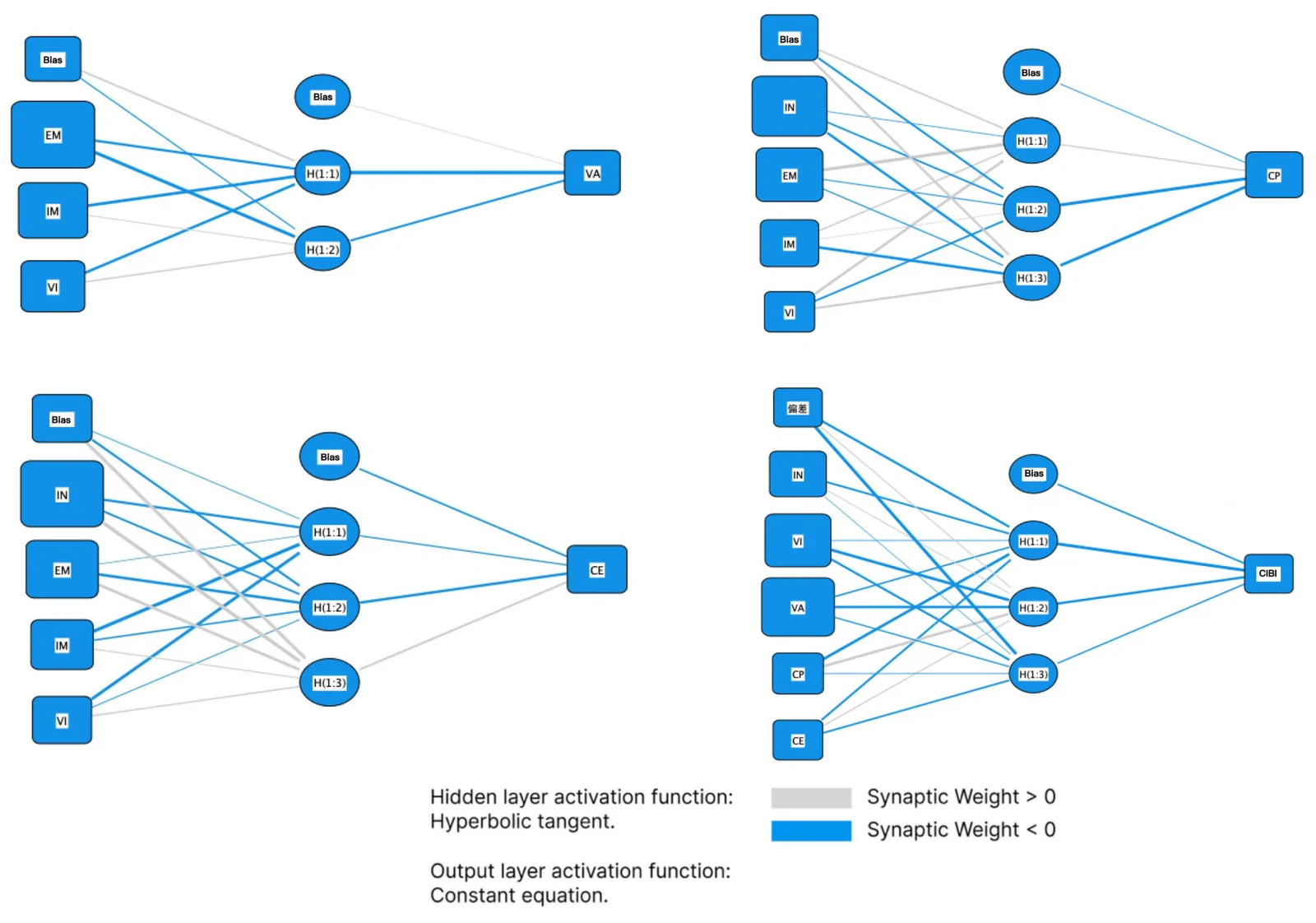
ANN model.

Structural equation modeling (SEM) is primarily used to test linear relationships among latent variables and therefore has limitations in capturing nonlinear and non-compensatory effects in complex behavioral mechanisms. Accordingly, recent studies have increasingly adopted a two-stage analytical strategy that integrates SEM with artificial neural network (ANN). This approach explains path relationships among variables while improving the model’ s predictive capability (Leong et al., 2013; Liébana-Cabanillas et al., 2017). Unlike SEM, ANN does not rely on strict parametric assumptions such as linearity, normality, and homoscedasticity, and can more effectively capture complex patterns in the formation of purchase intention (Loh et al., 2022; Xiong et al., 2022). Based on the SEM results, this study further conducted a supplementary analysis using the multilayer perceptron neural network in SPSS. Four ANN models were constructed according to the research model (Figure 8), with visual appeal (VA), consumer perceived arousal (CP), consumer engagement (CE), and consumer impulse buying intention (CIBI) specified as output variables. These models were used to examine the nonlinear predictive capability of the antecedent variables for the core outcome variables.

#### 4.3.2. Validation of ANN

In this study, the artificial neural network model was constructed using a multilayer perceptron (MLP). The dataset was first randomly divided into training and testing sets. To reduce the risk of overfitting, and following procedures commonly used in prior SEM–ANN studies, 90% of the sample was used for training and 10% for testing (Chong, 2013; Tan et al., 2014). The network adopted a single hidden-layer architecture, with the number of hidden nodes automatically determined by the system within a range of 1–50. For the activation functions, the hidden layer used the hyperbolic tangent function (tanh), whereas the output layer used the identity function. During model training, parameter updates were performed using the backpropagation algorithm. To further illustrate the computational mechanism of the ANN model, its basic structure can be expressed as Formula (1):

(1) $$y=f\left(\sum_{j=1}^{m}v_{j}f\left(\sum_{i=1}^{n}w_{ij}x_{i}+b_{j}\right)+b\right)$$

where $x_{i}$ denotes the input variable; $w_{ij}$ denotes the connection weight between the input and hidden layers; $v_{j}$ denotes the connection weight between the hidden and output layers; $b_{j}$ and *b* denote the bias terms; f(·) denotes the activation function; and *m* and *n* denote the number of hidden-layer nodes and input variables, respectively.

Predictive performance was evaluated using the mean root mean square error (RMSE) as the primary metric. To reduce the risk of overfitting, ten-fold cross-validation was employed for internal validation during model training. As shown in Table 10, the ANN models demonstrated moderate predictive capability, although the stability of test errors varied across models, particularly for Model D. Model A used EM, IM, and VI as input variables and VA as the output variable; its training RMSE ranged from 0.438 to 0.456 and its test RMSE from 0.297 to 0.581, with mean values of 0.447 and 0.456, respectively. Model B used IN, EM, IM, and VI as inputs and CP as the output, yielding training and test RMSE ranges of 0.409–0.440 and 0.398–0.499, with corresponding means of 0.426 and 0.443. Model C used IN, EM, IM, and VI as inputs and CE as the output; its training RMSE ranged from 0.444 to 0.468 and its test RMSE from 0.371 to 0.543, with mean values of 0.459 and 0.473, respectively. Model D used IN, VI, VA, CP, and CE as inputs and CIBI as the output. Its training RMSE ranged from 0.372 to 0.417, whereas its test RMSE ranged from 0.333 to 0.903, with mean values of 0.390 and 0.470, respectively. The relatively high variability in Model D’s test RMSE (Mean = 0.470, SD = 0.160) indicates limited predictive stability. Future research could improve model robustness through hyperparameter optimization, repeated cross-validation, and regularization. Overall, the ANN models showed useful predictive performance, but the results should be interpreted cautiously given the variability observed in test errors.

Overall, the ANN models demonstrated moderate predictive capability, although testing errors varied across models. Model D, in particular, exhibited substantial variability in testing RMSE. Accordingly, the current evidence is insufficient to draw definitive conclusions regarding the absence of overfitting or the models’ generalizability, and the results should therefore be interpreted with caution. Among the models, Model B achieved the lowest mean testing RMSE (Mean = 0.443, SD = 0.010), indicating the strongest predictive performance for consumer-perceived arousal (CP). In contrast, Model C yielded the highest mean testing RMSE (Mean = 0.473, SD = 0.008), suggesting greater complexity in predicting consumer engagement (CE).

#### 4.3.3. Sensitivity Analysis

Sensitivity analysis was further conducted to rank the relative importance of the input variables in the ANN models and identify the most influential predictors across models (Chong, 2013; Liébana-Cabanillas et al., 2017). As shown in Table 11, Model A ranked Empathy (100%) as the most important predictor, followed by Immersion (89.071%) and Vividness (84.153%). In Model B, Empathy (100%) again showed the highest importance, followed by Interactivity (98.603%), Immersion (68.161%), and Vividness (58.187%). In Model C, Interactivity (100%) was the most important predictor, followed by Empathy (94.588%), Vividness (65.360%), and Immersion (64.031%). In Model D, Vividness (100%) exhibited the highest importance, followed by Visual Attractiveness (88.559%), Interactivity (65.062%), Consumers’ Perceived Arousal (58.907%), and Consumer Engagement (49.421%). Overall, the relative importance of the antecedent variables varied considerably across models, indicating that the ANN analysis provides complementary insight into nonlinear predictive relationships that may not be fully captured by SEM.

To assess the consistency between the PLS-SEM and ANN results, this study compared the rankings of standardized path coefficients from the structural equation model with the normalized relative importance values from the artificial neural network (see Table 12). In Model A, with VA as the output variable, the rankings of EM→VA, IM→VA, and VI→VA were fully consistent across PLS-SEM and ANN, indicating strong agreement between the two methods in the visual appeal prediction model. In Model B, with CP as the output variable, the rankings of IN→CP, EM→CP, IM→CP, and VI→CP were also fully consistent, further confirming the stability of the results in the consumer perceived arousal prediction model. In Model C, with CE as the output variable, some differences emerged between the PLS-SEM and ANN results. IN→CE ranked first in both methods, indicating consistent evidence that interactivity is the most stable and critical predictor of consumer engagement. However, the relative rankings of EM→CE, IM→CE, and VI→CE varied slightly, suggesting that the effects of these variables on consumer engagement may be more complex under nonlinear prediction. In Model D, with CIBI as the output variable, the rankings of VI→CIBI, VA→CIBI, and CP→CIBI were fully consistent across PLS-SEM and ANN, whereas the rankings of IN→CIBI and CE→CIBI varied slightly. This suggests that the relative importance of antecedent variables in predicting consumer impulse buying intention may differ under a nonlinear analytical framework. Overall, the ANN results largely support the PLS-SEM findings, indicating that the proposed model has strong linear explanatory power and robust nonlinear predictive capability, thereby enhancing the robustness of the research conclusions.

## 5. Study 2. Qualitative Study Results

### 5.1. Grounded Theory-Informed Qualitative Analysis

Following completion of the Study 1 questionnaire survey, semi-structured interviews were conducted with participants recruited from the original survey sample. Participants were purposively selected based on variation in CIBI scores, including individuals with relatively low impulse buying tendencies, to enhance data heterogeneity and explanatory depth. All interviews were transcribed verbatim and analyzed using grounded theory-informed procedures involving open, axial, and selective coding. First, the 21 interview transcripts were coded line by line, yielding 36 open codes. Semantically related concepts were then consolidated into 12 axial coding categories. Finally, selective codes were derived from these axial categories and compared with the theoretical variables proposed in Chapter 2. Importantly, the qualitative analysis was not restricted to the pre-existing constructs examined in Study 1 but remained open to themes emerging directly from the interview data. As a result, the analysis identified both themes corresponding to the quantitative model and complementary themes extending beyond it. To enhance coding reliability, two coders independently conducted the initial coding and resolved discrepancies through discussion. No new core categories emerged after the 18th interview, and the final three interviews were used primarily to confirm the existing category structure. Accordingly, within the scope of this explanatory qualitative phase, the data were considered to have reached category saturation. The resulting selective codes showed substantial correspondence with several theoretical variables proposed in Chapter 2, thereby further supporting and enriching the explanatory scope of the original model (Table 13).

To assess the robustness of the category system, this study conducted a theoretical saturation test using staged theoretical sampling and the constant comparative method. The first 18 interviews were used to develop the preliminary category system, while the remaining three served as a validation set and were coded line by line to determine whether any new open codes or axial categories emerged. The additional interviews provided further semantic detail and contextual variation within the existing categories but did not generate new core categories or alter the relationships among them. These findings indicate that, within the current sample and stage of analysis, the major concepts had been sufficiently developed and the category system had reached stage-specific theoretical saturation (Hennink et al., 2017; Saunders et al., 2018). Nevertheless, additional concepts may emerge if future research extends the sample or examines different cultural or platform contexts. This saturation test further supports the stability of the qualitative category system and provides a foundation for integrating the findings of Study 1 and Study 2. Building on this analysis, a quantitative–qualitative joint display (Table 14) was developed to clarify the interconnections between the quantitative findings of Study 1 and the qualitative findings of Study 2.

To further clarify the interconnections between the two study phases, a quantitative–qualitative joint display (Table 14) was developed based on the coding results presented in Table 13. Study 2 was designed not only to corroborate the constructs examined in Study 1 but also to identify additional EC-DHA characteristics and consumer experience factors not fully captured by the quantitative model. Beyond themes corresponding to interactivity, empathy, immersion, vividness, and consumer experience, the interviews revealed several complementary themes outside the original model, including perceived professionalism, information persuasiveness, emotional contagion, and perceived convenience. Thus, the qualitative analysis both corroborated several quantitative relationships and provided additional insight into how consumer experience links EC-DHA characteristics to impulse buying intention. These findings were integrated with the quantitative paths and qualitative evidence presented in Table 14 and were subsequently discussed in Section 5.2 with reference to both significant and non-significant relationships. This integration helps explain the quantitative findings while extending the original model by identifying additional experiential factors not fully captured in Study 1.

### 5.2. Research Findings

The grounded theory-informed qualitative findings further revealed patterns of convergence, complementarity, and conditionality relative to the quantitative results of Study 1. As shown in Table 13, participants’ comments regarding the IN→CE pathway, such as “it can respond to my questions, which makes it feel interactive” and “it encourages me to interact,” are consistent with the significant positive relationship between interactivity and consumer engagement. This finding also aligns with prior research indicating that interactive experiences can enhance consumer engagement (Brodie et al., 2011; Hollebeek et al., 2014). For the EM→CP pathway, statements such as “its tone is relatively friendly” and “it speaks with enthusiasm, which also influences my emotions” provide qualitative support for the positive relationship between empathy and consumer-perceived arousal. Similarly, participants’ descriptions, including “the digital human looks attractive” and “it captures my attention,” are consistent with the significant VA→CIBI pathway and align with previous virtual streamer research showing that visual and emotional experiences can shape consumer responses (M. Li et al., 2022; Yu et al., 2024).

The qualitative findings also provide complementary explanations for several nonsignificant pathways. Specifically, the direct effects of IM→CIBI and EM→CIBI were not statistically significant. Participants typically described immersion as making them “easily become absorbed” and empathy as creating the impression that “it understands consumers quite well.” These accounts suggest that immersion and empathy may first strengthen attentional involvement and affective experience and subsequently influence purchase intention through other experiential mechanisms. Overall, Study 2 converges with several quantitative findings while also offering complementary explanations for nonsignificant pathways and identifying additional experiential factors beyond the original quantitative model.

## 6. Discussion

In the SEM–ANN comparison (Table 12), the results show a high degree of consistency between the two methods across most models while also revealing differences in the influence mechanisms of specific variables. In Model A, with VA as the output variable, and Model B, with CP as the output variable, the rankings based on PLS-SEM path coefficients were fully consistent with the ANN relative importance rankings. This indicates that the effects of EM, IM, VI, and IN on VA and CP were relatively stable. By contrast, Model C, with CE as the output variable, and Model D, with CIBI as the output variable, showed partial differences in variable rankings. This suggests that the formation mechanisms of CE and CIBI are more complex, and that some variables may play different roles in linear path analysis and nonlinear prediction. While PLS-SEM primarily reveals linear path relationships among variables, ANN further identifies the nonlinear importance of variables in predicting outcomes. Therefore, the partial differences between the SEM and ANN results enhance the explanatory depth of this study regarding the complex mechanisms through which EC-DHAs influence consumer experience and impulse buying intention.

However, the model’s overall explanatory power for the endogenous constructs was relatively modest. The $R^{2}$ values for consumer engagement (CE), visual appeal (VA), consumer-perceived arousal (CP), and consumer impulse buying intention (CIBI) were 0.094, 0.105, 0.161, and 0.219, respectively, indicating that the EC-DHA characteristics and consumer experience variables included in the model explained only a limited proportion of the variance in these constructs. In addition, some statistically significant structural paths exhibited relatively small standardized coefficients, such as IN→CIBI (β = 0.112) and VI→CE (β = 0.101). Accordingly, statistical significance should not be interpreted as evidence of strong practical effects. Rather, the findings suggest that EC-DHA characteristics are among several factors associated with consumer experience and impulse buying intention, rather than dominant determinants of consumer behavior.

The integrated “EC-DHA characteristics–consumer experience–impulse buying intention” model developed in this study aims to examine the mechanisms through which DHAs influence consumers’ impulse buying intention. Specifically, the study focuses on the relationships among DHAs’ interactive performance, technological perception, consumer experience, emotional responses, and impulse buying intention in e-commerce live-streaming contexts (Table 15).

This study employed a mixed-methods approach to systematically examine the mechanisms and boundary conditions through which e-commerce digital human avatars (EC-DHAs) are associated with consumers’ impulse buying intention in livestream shopping. By integrating DHA characteristics (IN, EM, IM, and VI), consumer experiential and psychological responses (VA, CP, and CE), and the behavioral outcome (CIBI), the study combined PLS-SEM and artificial neural network (ANN) analyses to provide complementary evidence on structural relationships and nonlinear predictive patterns. Interactivity (IN), vividness (VI), visual appeal (VA), consumer-perceived arousal (CP), and consumer engagement (CE) were all significantly and positively associated with consumer impulse buying intention (CIBI). However, the corresponding path coefficients were generally modest, indicating that statistical significance should not be interpreted as evidence of strong practical effects. Rather, these findings suggest statistically meaningful associations and reveal differences in the relative patterns through which the examined factors relate to impulse buying intention. The model also showed limited overall explanatory power. The $R^{2}$ value for CIBI was 0.219, while those for CP, VA, and CE were 0.161, 0.105, and 0.094, respectively. These values indicate that the current model explains only a limited proportion of the variance in consumer responses. In particular, approximately 78% of the variance in CIBI remains unexplained, suggesting that additional factors, such as price promotions, product-category involvement, brand equity, individual impulsivity, platform trust, and other contextual variables, may also contribute to impulse buying intention. Future research could incorporate these factors to assess their incremental explanatory value. This interpretation is consistent with prior research on impulse buying intention in livestream e-commerce (Lee & Chen, 2021; M. Li et al., 2022). Regarding consumer experiential and psychological responses, the path coefficient between empathy (EM) and consumer-perceived arousal (CP) was relatively large, indicating a comparatively strong association between these constructs in the present sample. Prior research on virtual streamers and digital humans similarly suggests that affective interaction can enhance immersion, trust, and emotional arousal, thereby increasing purchase intention (Lou et al., 2023; Miao et al., 2022). Visual appeal (VA) was also significantly and positively associated with CIBI, suggesting that the appearance, image style, and visual presentation of digital humans may be related to stronger impulse buying intention. By contrast, the direct relationships of empathy (EM) and immersion (IM) with CIBI were nonsignificant, whereas significant indirect associations emerged through visual appeal, perceived arousal, and consumer engagement. From an S-O-R perspective, this pattern indicates that different EC-DHA stimuli may operate through distinct experiential pathways rather than affecting consumer responses in a uniform manner. H1a was also unsupported (β = 0.034, *p* = 0.431). Interactivity primarily reflects bidirectional communication, immediate feedback, and participation control, whereas visual appeal depends more strongly on the digital human’s appearance and visual presentation. Interactivity may therefore be more directly associated with consumer arousal and engagement than with visual appeal. More broadly, interactivity and vividness represent relatively immediate informational cues that consumers can directly perceive and process, whereas empathy and immersion may operate primarily through internal experiential states, such as visual evaluation, affective activation, and participatory involvement, before becoming associated with impulse buying intention.

The findings further indicate that the stimulus–response relationship within the S-O-R framework is not a simple process of direct transmission but depends on whether and how different stimuli activate experiential states at the Organism level. Consumer engagement may therefore serve as an important mechanism linking digital interaction experiences to purchase intention, with higher engagement associated with greater viewing involvement and stronger impulse buying intention (Brodie et al., 2011; Hollebeek et al., 2014). Among the three consumer experience variables, visual appeal (VA) exhibited the strongest path coefficient with impulse buying intention, suggesting a comparatively strong association between the visual presentation and attractiveness of DHAs and consumers’ impulse buying intention (Lou et al., 2023; Miao et al., 2022). Overall, DHA design should integrate visual appearance, affective expression, information presentation, and interactive participation. By optimizing the livestream environment and product presentation, platforms may enhance consumers’ perceived arousal and engagement, which may in turn strengthen impulse buying intention (Brodie et al., 2011; Hollebeek et al., 2014; M. Li et al., 2022).

Second, the comparison between the PLS-SEM and ANN results demonstrates both methodological complementarity and theoretical relevance. PLS-SEM captures average linear relationships among variables, whereas the relative importance derived from ANN suggests that the relationships between EC-DHA cues and consumer experiences may not follow uniformly linear patterns. Some variables exhibited only moderate coefficients in the structural model but showed relatively high predictive importance in the ANN analysis, indicating that consumer responses to EC-DHA stimuli may involve nonlinear or conditional patterns. Accordingly, within the EC-DHA context, the stimulus–experience relationships specified by the S-O-R framework should not be interpreted solely as a simple linear accumulation process. Instead, attention should be given to the differentiated roles of specific stimuli within cue combinations and the formation of consumer experiences. However, ANN results primarily indicate predictive importance and should not, on their own, be interpreted as evidence of causal mechanisms.

Third, the qualitative findings were broadly consistent with the quantitative results from the PLS-SEM and ANN analyses. A particularly noteworthy finding is that empathy (EM) and immersion (IM) did not have significant direct effects on consumer impulse buying intention (CIBI). Accordingly, H2d and H3d were not supported, indicating that no significant direct relationships between EM, IM, and CIBI were observed in the present context. When considered alongside their significant indirect effects, this pattern is consistent with the alternative theoretical possibility proposed during hypothesis development: empathy and immersion may be associated with impulse buying intention primarily through visual evaluation, perceived arousal, and engagement-related experiences. However, because competing hypotheses were not preregistered, this interpretation should be treated as a cautious post hoc explanation rather than confirmation of a prespecified indirect-effect hypothesis. Future research could employ preregistered designs to compare direct and indirect pathways more rigorously. From a practical perspective, the findings suggest that EC-DHAs may not stimulate impulse buying through anthropomorphic features or immersive experiences alone. Rather, emotional and immersive experiences may need to be translated into clear product value, immediate purchase-related cues, and mechanisms that encourage interaction and participation. The findings also support the multidimensional conceptualization of the Organism component adopted in this study. Consumers may not develop purchase impulses immediately after exposure to EC-DHA stimuli but may instead undergo distinct internal experiential processes, including visual evaluation, affective activation, and participatory involvement. Thus, the qualitative findings not only complement the quantitative results but also provide a context-specific explanation of how EC-DHA stimuli may be transformed into internal experiences and subsequently associated with behavioral responses within the S-O-R framework.

Overall, the key finding of this study is that EC-DHA characteristics are not uniformly associated with consumer impulse buying intention but operate through distinct pathways. Interactivity and vividness show direct associations with impulse buying intention, whereas empathy and immersion appear to operate primarily through consumers’ internal experiential states. In particular, visual appeal and consumer-perceived arousal emerge as important experiential mechanisms linking EC-DHA characteristics to impulse buying intention, suggesting that external stimuli may influence behavioral tendencies through consumers’ visual evaluations and affective activation. At the same time, impulse buying intention is likely shaped by additional individual and contextual factors beyond those included in the present model. Accordingly, this study emphasizes the differentiated mechanisms through which EC-DHA characteristics are associated with impulse buying intention rather than presenting the model as a comprehensive explanation of consumer behavior.

### 6.1. Theoretical Contributions

**Figure 9:**
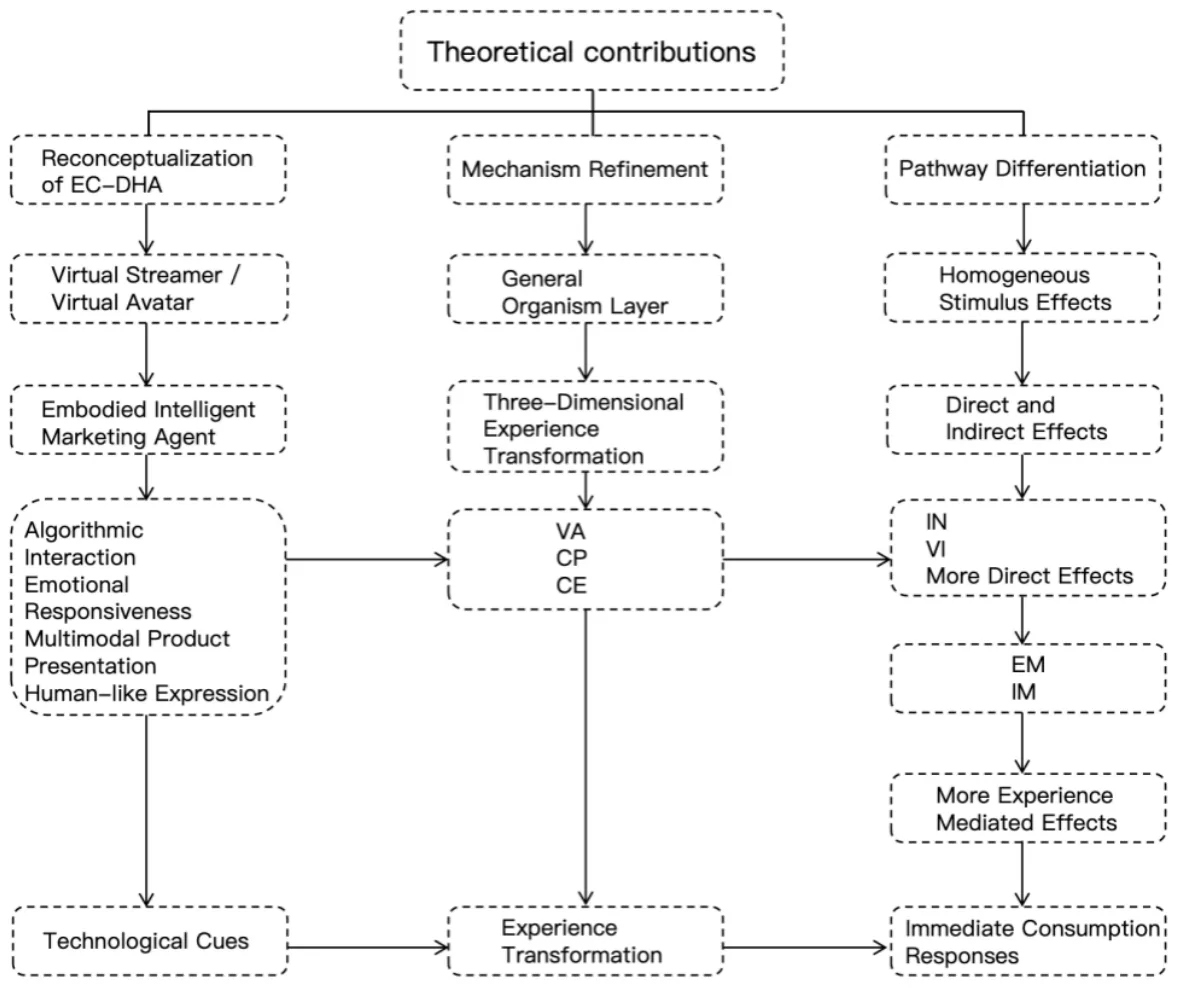
EC-DHA Theoretical Contribution Framework.

This study makes three theoretical contributions to the literature on impulse buying in EC-DHA livestreaming contexts (Figure 9).

First, rather than merely applying the S-O-R model to the digital human livestreaming context, this study reconceptualizes EC-DHAs as embodied intelligent marketing avatars. Unlike traditional human streamers, conventional virtual streamers, or static product-display interfaces, EC-DHAs integrate multiple capabilities, including algorithmic interaction, anthropomorphic expression, emotional responsiveness, and multimodal product presentation. Accordingly, EC-DHAs function not merely as information-delivery tools but as intelligent marketing intermediaries that connect product information, consumer experience, and immediate consumption decisions in livestream shopping (M. Li et al., 2022; Lou et al., 2023; Miao et al., 2022). This perspective extends current understandings of streamers and virtual avatars in livestream e-commerce by shifting attention from appearance-based anthropomorphism to their capacity to shape consumer experiences as intelligent interactive avatars.

Second, this study provides a consumer experience-based contextual refinement of the Organism component within the S-O-R framework. The classic S-O-R framework conceptualizes the organism as the internal psychological state linking external environmental stimuli to behavioral responses primarily through affective and cognitive processes (Eroglu et al., 2003; Mehrabian & Russell, 1974). Prior livestream e-commerce research has also employed visual appeal, perceived arousal, and consumer engagement to explain how environmental cues are associated with impulse buying (Yang et al., 2022). Accordingly, the theoretical contribution of this study does not lie in introducing these constructs for the first time or merely combining them. Rather, it lies in situating them within the EC-DHA livestreaming context and conceptualizing them as three complementary dimensions of internal experience: visual evaluation, affective activation, and participatory involvement. Specifically, visual appeal reflects consumers’ aesthetic evaluations of the EC-DHA’s appearance, interface, and product presentation (Deng & Poole, 2012); consumer-perceived arousal captures the immediate affective activation elicited by digital human interaction and the livestream environment (Mehrabian & Russell, 1974; Yang et al., 2022); and consumer engagement reflects the cognitive, affective, and behavioral involvement that develops during viewing and interaction (Hollebeek et al., 2014). Building on this conceptualization, the study examines how different EC-DHA characteristics are associated with distinct direct and indirect pathways through these experiential dimensions. Thus, rather than simply adding established consumer response variables, this study offers a structured explanation of how EC-DHA stimuli in AI-driven livestream e-commerce are associated with consumers’ internal experiences and impulse buying intention through visual evaluation, affective activation, and participatory involvement. In doing so, it further clarifies the context-specific manifestation of the Organism component within the S-O-R framework. However, given the relatively limited $R^{2}$ values of the Organism variables, the contribution of this study lies primarily in identifying and organizing distinct consumer experience dimensions and their differentiated pathways in the EC-DHA context, rather than claiming that the model provides a comprehensive explanation of consumers’ internal experiences.

Third, this study reveals differentiated relationships between distinct EC-DHA characteristics and impulse buying intention. The findings indicate that interactivity and vividness are more directly associated with impulse buying intention, whereas empathy and immersion do not necessarily translate into purchase impulses through direct pathways. Consumers may therefore be unlikely to make an immediate purchase solely because a digital human appears human-like, communicates warmly, or creates an immersive livestreaming experience. Instead, empathy and immersion appear to function primarily as antecedent experiential conditions that enhance receptivity to livestream content, viewing involvement, and emotional connection. These experiences may subsequently be associated with impulse buying intention through visual appeal, perceived arousal, and consumer engagement. Overall, the results suggest that EC-DHA characteristics do not operate through uniform pathways; rather, empathy and immersion appear to exert their roles mainly through consumer experience mechanisms. This differentiation further clarifies the stimulus–experience–response processes underlying EC-DHA-enabled livestream commerce. Accordingly, this study extends research on digital human livestreaming, intelligent marketing avatars, and online impulse buying intention by offering a more nuanced theoretical explanation of how technological cues are transformed through consumer experience into immediate consumption responses.

### 6.2. Practical Contributions

At the practical level, this study offers three implications for the design and operation of EC-DHAs based on the empirical findings. First, platforms may place greater emphasis on the emotional expression and visual experience design of digital humans. Empathy (EM) was significantly associated with visual appeal (VA), consumer-perceived arousal (CP), and consumer engagement (CE), with the strongest relationship observed for CP. Accordingly, in specific livestreaming contexts, platforms may consider the digital human’s visual style, dynamic product presentation, explanatory visuals, and scene design in relation to brand positioning, product type, and target user characteristics, rather than setting design priorities based on a single path coefficient. Second, platforms may develop EC-DHAs with role characteristics aligned with specific brand positions and consumption contexts. Based on brand identity, product characteristics, and target user preferences, e-commerce platforms could design digital human personas with clearly defined roles, such as professional beauty consultants, approachable lifestyle recommenders, or trend-oriented virtual streamers. Such role-based design may help strengthen consumer trust, involvement, and shopping interest. Establishing a branded digital human identity may also support the continued use of DHAs in livestream marketing, product recommendation, and consumer experience optimization (Chaihanchanchai et al., 2026). Third, the conversational capabilities of EC-DHAs may provide a useful design direction for improving access to product information and enhancing consumer participation. However, their effectiveness is likely to depend on the combined influence of promotional conditions, product categories, brand-related factors, and individual consumer characteristics.

Furthermore, different stakeholders may adopt tailored strategies based on these findings. Livestream platform designers may optimize product displays, interaction entry points, and information feedback mechanisms to enhance consumers’ visual experience and sense of participation. EC-DHA developers may focus on improving visual presentation, natural expression, emotional feedback, and interactive responsiveness so that digital humans can more effectively support consumer experience. Online retailers may select EC-DHA appearances and presentation styles that align with brand positioning, product characteristics, and target consumers, using them as complementary tools to enhance the shopping experience and support impulse buying intention. Digital marketers may combine vivid product presentation, interactive communication, and emotional expression rather than relying solely on empathy or immersion to directly stimulate purchasing. Consumer protection policymakers, meanwhile, should prioritize the transparency of product, pricing, and promotional information in digital human livestreaming and seek to prevent excessive urgency cues or emotional stimulation from undermining consumers’ autonomous decision-making.

In summary, the value of EC-DHAs lies not only in enhancing consumer experience and purchase intention but also in supporting consumer well-being and responsible design. While optimizing visual presentation, emotional expression, and interactive experiences, platforms should avoid excessive persuasion through urgency cues or emotional stimulation and instead support more deliberate decision-making through appropriate purchase prompts and mechanisms that preserve user autonomy. The visual design, emotional expression, and interactive features of digital humans may be associated with consumer engagement, perceived arousal, and impulse buying intention. However, these design factors constitute only part of the broader consumer decision environment, and their effects may also depend on price promotions, product-category involvement, brand equity, and other individual and contextual factors. Importantly, the model explains 21.9% of the variance in CIBI, indicating that EC-DHA design characteristics account for only a limited proportion of differences in consumer impulse buying intention. Accordingly, the proposed design strategies should be understood as incremental approaches for improving consumer experience and purchase intention rather than as interventions expected to produce substantial increases in actual conversion rates. Moreover, because this study measured impulse buying intention rather than actual purchasing behavior, the findings cannot be used to estimate specific improvements in conversion rates. Future research and platform evaluations could incorporate experimental tests, clickstream data, add-to-cart behavior, and actual purchase records to assess the real-world conversion effects and cost-effectiveness of alternative EC-DHA design strategies.

### 6.3. Methodological Contributions

In terms of methodological contributions, this study integrates PLS-SEM and ANN as complementary analytical approaches, thereby addressing the limitations of relying on a single method to explain consumer impulse buying intention and enhancing the robustness of the analysis. PLS-SEM is used to examine linear structural relationships, whereas ANN is employed to identify potential nonlinear predictive relationships and assess the relative importance of key predictors. This combination provides a more comprehensive analytical framework for examining complex consumer behavior and strengthens the reliability of the findings (Chong, 2013; Dul et al., 2020; Hair et al., 2019). Building on these quantitative analyses, the study further triangulates the findings with qualitative interviews and grounded theory-informed analysis to contextualize the relationships between EC-DHA characteristics, including visual appearance, emotional expression, information presentation, and interactive experience, and consumers’ visual appeal, perceived arousal, and engagement. Qualitative findings related to visual attraction, emotional arousal, promotional stimuli, and immersive shopping atmospheres complement and corroborate key patterns identified through the PLS-SEM and ANN analyses. Taken together, these findings establish a coherent evidence chain linking “EC-DHA cues–consumer experience–impulse buying intention” and provide a replicable mixed-methods framework and analytical procedure for future research on DHAs and consumer impulse buying intention.

### 6.4. Limitation and Future Research

Although this study makes theoretical, methodological, and practical contributions, several limitations should be acknowledged. First, although the qualitative sample included participants from diverse age groups and occupational backgrounds, the study focused primarily on the overall mechanisms linking EC-DHA characteristics to impulse buying intention and did not identify which consumer groups may be more responsive to these influences. Individual differences, including age, livestream shopping experience, trait impulsivity, and product involvement, may represent important boundary conditions. In addition, omitted factors such as price promotions, product involvement, brand equity, and platform trust may account for part of the unexplained variance. Future research should examine the moderating or incremental roles of these factors. Second, this study combined PLS-SEM and ANN to examine linear structural relationships and nonlinear predictive patterns. However, the relatively large variation in the test RMSE of Model D suggests limited predictive stability and warrants caution when interpreting its generalizability. Future studies could improve predictive robustness by using larger samples, repeated cross-validation, hyperparameter optimization, regularization, and independent validation datasets. Finally, this study employed purposive sampling and included only Chinese consumers with prior experience of EC-DHAs or livestream shopping. The findings therefore do not provide probability-based representation of the broader Chinese consumer population and should not be directly generalized to other cultural contexts. Moreover, JD.com’s “Caixiao Dongge” AI digital human livestream served as the sole standardized research context. The S-O-R relationships examined in this study are therefore specific to this platform and case. Because “Caixiao Dongge” is modeled on a well-known corporate founder, consumers’ evaluations of empathy and vividness may also be influenced by brand trust and familiarity with the individual. The findings should therefore not be generalized directly to generic, hyper-realistic, or anime-style EC-DHAs. In addition, the cross-sectional survey design captures statistical associations rather than causal relationships. The Organism variables were measured through self-reports collected after exposure to a standardized prerecorded stimulus and thus represent subjective post-exposure experiences rather than momentary psychological states during actual interaction. This measurement approach may constrain construct validity and ecological interpretation. Future research could further distinguish immersive environmental cues from internal immersive experiences and test the model across platforms, cultures, and real-time livestream settings. Integrating real-time experiential measures with actual purchasing data would also allow more rigorous assessment of external validity, causal relationships, and ecological validity.

## 7. Conclusions

This study examined the mediating mechanisms linking EC-DHA characteristics, consumer experience, and consumer impulse buying intention in the context of Chinese livestream e-commerce and identified several theoretical and practical implications. First, vividness (VI) was significantly and positively associated with consumer impulse buying intention and showed a comparatively prominent role among the stimulus characteristics examined. This finding suggests that the dynamic presentation, contextual display, and information delivery of EC-DHAs may be associated with consumers’ shopping experiences and immediate purchase tendencies. Second, visual appeal (VA) was significantly and positively associated with consumer impulse buying intention, indicating that the visual design of digital humans, interface presentation, and product display may contribute to more positive visual experiences and stronger purchase tendencies. Third, consumer-perceived arousal (CP) and consumer engagement (CE) emerged as important experiential pathways linking EC-DHA characteristics to impulse buying intention. Although the direct relationships of empathy and immersion with impulse buying intention were nonsignificant, both constructs showed significant indirect associations through consumer experience variables. This pattern suggests that the relationships between EC-DHA characteristics and impulse buying intention may operate through multiple experiential pathways. Fourth, the mixed-methods design provided complementary evidence from different analytical perspectives. PLS-SEM, ANN, and qualitative analysis contributed insights into structural relationships, nonlinear predictive patterns, and consumers’ experiential interpretations, respectively, thereby supporting a more comprehensive understanding of consumer responses in EC-DHA livestream shopping. Fifth, from a practical perspective, e-commerce platforms and EC-DHA designers may consider vivid presentation, visual appearance, interactive feedback, and emotional expression as relevant design directions for improving livestream shopping experiences. However, because this study is based on cross-sectional data, the findings reflect statistical associations and mediation patterns rather than causal relationships. Moreover, the study measured impulse buying intention rather than actual purchasing behavior, and the model demonstrated relatively limited explanatory power. Overall, EC-DHA characteristics exhibit differentiated direct and indirect associations with impulse buying intention, with consumers’ visual, affective, and engagement-related experiences constituting important experiential pathways.

## Figures and Tables

**Table 1:** Review of Research on E-Commerce Digital Human Avatars (EC-DHAs).

Source	Theoretical Foundation/Perspective	Research Context	Key Antecedents and Mechanisms	Outcome
Gao et al. (2023)	S-O-R/Presence Perspective	Virtual streamer-based livestream e-commerce	Likability, perceived aliveness, and responsiveness → social presence and telepresence	Purchase intention
Wu et al. (2023)	Social Presence/Experiential Value Perspective	Virtual livestream e-commerce	Socialness → social presence → utilitarian and hedonic experiential value	Experiential value
Hu and Ma (2023)	Language Expectancy Theory	Virtual streamer-based livestream e-commerce	Sensory language → language expectancy violation	Purchase intention
Yao et al. (2024)	Stereotype Content Model	Digital human livestream e-commerce	Social-oriented vs. task-oriented language → perceived warmth and competence	Purchase intention
Y. Zhang and Wang (2025)	Warmth–Competence/ Stereotype Perspective	Commercial virtual streamer context	Virtual streamer appearance → perceived warmth and competence	Purchase intention
C. Li and Huang (2024)	Avatar Theory	Virtual streamer e-commerce	Multidimensional anthropomorphism → cognitive trust	Purchase intention
Xie et al. (2024)	Meaning Transfer Theory	Comparison of human-driven virtual streamers and human streamers	Streamer type → consumer empathy	Brand forgiveness
Yan et al. (2025)	Mental Imagery Perspective	Comparison of virtual and human streamers	Streamer type × product type → mental imagery quality	Purchase intention
Qin and Liu (2025)	Cognition–Affect–Behavior Model/Psychological Contract Perspective	Virtual streamer-based livestream e-commerce	Competence, interaction quality, and warmth → psychological contract	Purchase intention
Xu et al. (2025)	Social Identity Theory/Experiential Value Theory	AI virtual streamer context	Personalization, human-likeness, and system/content quality → parasocial interaction and experiential value	Brand image
H. Liu et al. (2025)	Social Presence/Perceived Value Perspective	AI-generated virtual streamer e-commerce	Interaction type → social presence and perceived value	Purchase intention
Shui et al. (2025)	Trust Perspective	AI virtual streamer-based livestream e-commerce	Appearance characteristics, competence characteristics, and contextual fit → consumer trust	Purchase intention
Gao et al. (2025)	Stereotype Content Model	Virtual streamer-based livestream e-commerce	Virtual streamer coolness → perceived warmth and competence	Purchase intention
Gu et al. (2026)	Affect–Cognition Perspective	AI-generated virtual streamer e-commerce	Virtual streamer characteristics → attractiveness and cognitive fluency	Patronage intention
Z. Liu et al. (2026)	Affect–Cognition Decision-Making Perspective	Virtual and human streamer-based livestream e-commerce	Streamer type → affective and cognitive decision-making processes	Impulse buying intention

Source: Authors’ own compilation.

**Table 2:** Construct conceptualization and item.

Variable	Item	Measurement Item	Reference
Interactivity (IN)	IN1	The EC-DHA responds to my questions in a timely manner.	Y. Liu (2003)
	IN2	I can communicate with the EC-DHA in a two-way manner rather than merely receiving information passively.	
	IN3	I can obtain recommendations from the EC-DHA that match my needs.	
	IN4	The communication process with the EC-DHA is smooth.	
Empathy (EM)	EM1	When I hesitate to make a purchase, the EC-DHA’s response makes me feel reassured.	Davis (1983)
	EM2	I feel that the EC-DHA pays attention to my personal preferences.	
	EM3	The EC-DHA patiently answers my questions.	
	EM4	I feel respected when interacting with the EC-DHA.	
Immersion (IM)	IM1	When watching the EC-DHA, I easily ignore my surroundings.	Jennett et al. (2008)
	IM2	I unconsciously devote more attention during the shopping process.	
	IM3	During the interaction, I feel that time passes quickly.	
	IM4	Interacting with the EC-DHA gives me a sense of being there.	
Vividness (VI)	VI1	Interacting with the EC-DHA provides me with a rich sensory experience.	Steuer (1992)
	VI2	Watching the EC-DHA introduce products helps me better understand product information.	
	VI3	I think the EC-DHA presents content in a vivid manner.	
	VI4	The EC-DHA’s presentation styles make the products feel more realistic.	
Visual Appeal (VA)	VA1	Compared with traditional shopping interfaces, the EC-DHA is more effective in attracting my interest.	Cyr et al. (2006)
	VA2	The presentation style of the EC-DHA is visually appealing.	
	VA3	I am satisfied with the interface or visual style of the EC-DHA.	
	VA4	The image design of the EC-DHA is aesthetically pleasing.	
Consumer Perceived Arousal (CP)	CP1	The EC-DHA’s presentation generates strong purchase interest in me.	Mehrabian and Russell (1974)
	CP2	I feel excited when interacting with the EC-DHA.	
	CP3	Interacting with the EC-DHA makes me pay more attention to the product.	
	CP4	After the EC-DHA introduces a product, I become more curious about it.	
Consumer Engagement (CE)	CE1	When watching the EC-DHA, I obtain more product information.	Dessart et al. (2015)
	CE2	I continue to pay attention to the EC-DHA’s content during the shopping process.	
	CE3	I feel relaxed when interacting with the EC-DHA.	
Consumer Impulse Buying Intention (CIBI)	CIBI1	Sometimes I think about purchasing products without prior planning.	Rook (1987)
	CIBI2	I develop the intention to purchase products because of temporary interest.	
	CIBI3	When I see a product that interests me, I feel a strong impulse to buy it.	
	CIBI4	If product information or promotional content is sufficiently attractive, I develop the intention to place an order immediately.	

Note: The consumer-perceived arousal (CP) items were theoretically grounded in the arousal dimension of the PAD framework and contextually adapted to the EC-DHA livestream shopping context, rather than directly replicating the classical adjective-based PAD arousal scale.

**Table 3:** Cronbach’s α, composite reliability and average variance extracted.

Variables	Cronbach’s Alpha	Composite Reliability	AVE	Items	VIF	Factor Loading
CE	0.816	0.826	0.730	CE1	1.666	0.857
				CE2	2.085	0.863
				CE3	1.835	0.842
CIBI	0.904	0.907	0.777	CIBI1	2.030	0.842
				CIBI2	3.107	0.903
				CIBI3	3.711	0.909
				CIBI4	2.917	0.871
CP	0.914	0.918	0.794	CP1	2.486	0.876
				CP2	3.147	0.905
				CP3	3.590	0.915
				CP4	2.793	0.867
EM	0.933	0.936	0.832	EM1	3.728	0.906
				EM2	4.162	0.926
				EM3	4.228	0.935
				EM4	2.837	0.881
IM	0.875	0.879	0.729	IM1	2.725	0.869
				IM2	3.147	0.890
				IM3	2.324	0.871
				IM4	1.691	0.781
IN	0.824	0.828	0.655	IN1	1.599	0.776
				IN2	1.894	0.808
				IN3	2.114	0.837
				IN4	1.887	0.814
VA	0.937	0.945	0.840	VA1	3.332	0.906
				VA2	4.790	0.941
				VA3	4.739	0.934
				VA4	3.380	0.884
VI	0.904	0.914	0.776	VI1	2.888	0.869
				VI2	3.688	0.918
				VI3	3.112	0.908
				VI4	2.147	0.827

**Table 4:** Fornell–Larcker criterion.

	CE	CIBI	CP	EM	IM	IN	VA	VI
CE	0.854							
CIBI	0.220	0.882						
CP	0.146	0.248	0.891					
EM	0.186	0.139	0.235	0.912				
IM	0.104	0.094	0.166	−0.069	0.854			
IN	0.186	0.172	0.230	−0.027	0.037	0.809		
VA	−0.019	0.280	0.120	0.221	0.157	0.030	0.916	
VI	0.118	0.306	0.162	0.089	0.036	−0.024	0.185	0.881

**Table 5:** HTMT values.

	CE	CIBI	CP	EM	IM	IN	VA	VI
CE								
CIBI	0.249							
CP	0.165	0.269						
EM	0.207	0.152	0.251					
IM	0.124	0.106	0.188	0.076				
IN	0.219	0.195	0.260	0.039	0.061			
VA	0.027	0.304	0.127	0.232	0.172	0.068		
VI	0.135	0.337	0.174	0.094	0.054	0.037	0.195	

**Table 6:** Coefficient of Determination ($R^{2}$) and Predictive Correlation ($Q^{2}$) of the Model.

Latent Variable	$R^{2}$ Value	$Q^{2}$ Value
Consumer Engagement (CE)	0.094	0.075
Consumer Impulse Buying Intention (CIBI)	0.219	0.129
Consumers’ Perceived Arousal (CP)	0.161	0.143
Visual Attractiveness (VA)	0.105	0.090

**Table 7:** A summary of the results of hypotheses testing.

Hypothesis	Original Sample (O)	Mean (M)	STDEV	T Statistics	*p* Values	Statistical Support
CE→CIBI	0.154	0.153	0.045	3.378	0.001 **	Supported
CP→CIBI	0.132	0.132	0.048	2.727	0.006 **	Supported
EM→CE	0.190	0.190	0.045	4.185	0.000 ***	Supported
EM→CIBI	0.015	0.015	0.045	0.342	0.732	Unsupported
EM→CP	0.241	0.241	0.041	5.869	0.000 ***	Supported
EM→VA	0.220	0.220	0.044	5.044	0.000 ***	Supported
IM→CE	0.107	0.108	0.043	2.501	0.012 *	Supported
IM→CIBI	0.011	0.011	0.042	0.266	0.790	Unsupported
IM→CP	0.169	0.171	0.043	3.937	0.000 ***	Supported
IM→VA	0.166	0.168	0.044	3.735	0.000 ***	Supported
IN→CE	0.190	0.192	0.046	4.091	0.000 ***	Supported
IN→CIBI	0.112	0.112	0.043	2.625	0.009 **	Supported
IN→CP	0.233	0.235	0.041	5.649	0.000 ***	Supported
IN→VA	0.034	0.034	0.043	0.787	0.431	Unsupported
VA→CIBI	0.216	0.217	0.045	4.839	0.000 ***	Supported
VI→CE	0.101	0.103	0.042	2.431	0.015 *	Supported
VI→CIBI	0.228	0.228	0.043	5.308	0.000 ***	Supported
VI→CP	0.140	0.140	0.044	3.210	0.001 **	Supported
VI→VA	0.160	0.162	0.043	3.740	0.000 ***	Supported

Note: IN: Interactivity; EM: Empathy; IM: Immersion; VI: Vividness; VA: Visual Attractiveness; CP: Consumers’ Perceived Arousal; CE: Consumer Engagement; CIBI: Consumer Impulse Buying Intention. *** p<0.001, ** p<0.01, * p<0.05. Hypothesis support indicates statistical significance and should not be interpreted as evidence of effect magnitude or practical importance.

**Table 8:** Indirect effects.

Paths	Original Sample (O)	Sample Mean (M)	Standard Deviation (STDEV)	T Statistics (|O/STDEV|)	*p* Values	Results
EM→CP→CIBI	0.032	0.032	0.013	2.489	0.013 *	Support
VI→VA→CIBI	0.035	0.035	0.012	2.799	0.005 **	Support
IM→CP→CIBI	0.022	0.022	0.010	2.241	0.025 *	Support
EM→CE→CIBI	0.029	0.029	0.012	2.453	0.014 *	Support
IN→CP→CIBI	0.031	0.031	0.013	2.440	0.015 *	Support
IM→CE→CIBI	0.016	0.017	0.009	1.925	0.054	Unsupported
IN→CE→CIBI	0.029	0.029	0.012	2.491	0.013 *	Support
VI→CP→CIBI	0.018	0.018	0.009	2.023	0.043 *	Support
VI→CE→CIBI	0.016	0.016	0.009	1.829	0.067	Unsupported
EM→VA→CIBI	0.047	0.048	0.014	3.455	0.001 **	Support
IM→VA→CIBI	0.036	0.036	0.012	2.874	0.004 **	Support
IN→VA→CIBI	0.007	0.007	0.010	0.751	0.453	Unsupported

Note: ** p<0.001, * p<0.01.

**Table 9:** Mediation Analysis Results and VAF.

Relationship	Direct Effect	Total Indirect Effect	Total Effect	VAF (%)	Mediation Type
EM→CIBI	0.015	0.108	0.124	87.10	Full mediation
IM→CIBI	0.011	0.074	0.086	86.05	Full mediation
IN→CIBI	0.112	0.067	0.179	37.43	Partial mediation
VI→CIBI	0.228	0.069	0.296	23.31	Partial mediation

**Table 10:** RMSE values for Models A, B, C, and D.

**ANN**	**Model A**		**Model B**	
	**Input: EM, IM, VI**		**Input: IN, EM, IM, VI**	
	**Output: VA**		**Output: CP**	
	**Training**	**Testing**	**Training**	**Testing**
ANN1	0.442	0.464	0.423	0.436
ANN2	0.453	0.369	0.427	0.444
ANN3	0.453	0.474	0.424	0.426
ANN4	0.446	0.420	0.427	0.398
ANN5	0.442	0.581	0.425	0.487
ANN6	0.438	0.477	0.440	0.499
ANN7	0.444	0.508	0.439	0.421
ANN8	0.447	0.456	0.409	0.445
ANN9	0.451	0.509	0.413	0.402
ANN10	0.456	0.297	0.437	0.475
Mean	0.447	0.456	0.426	0.443
SD	0.006	0.079	0.010	0.034
**ANN**	**Model C**		**Model D**	
	**Input: IN, EM, IM, VI**		**Input: IN, VI, VA, CP, CE**	
	**Output: CE**		**Output: CIBI**	
	**Training**	**Testing**	**Training**	**Testing**
ANN1	0.452	0.472	0.383	0.390
ANN2	0.450	0.451	0.394	0.482
ANN3	0.465	0.463	0.385	0.333
ANN4	0.468	0.469	0.417	0.439
ANN5	0.459	0.522	0.381	0.903
ANN6	0.464	0.416	0.372	0.498
ANN7	0.461	0.371	0.380	0.408
ANN8	0.465	0.534	0.405	0.470
ANN9	0.457	0.489	0.388	0.385
ANN10	0.444	0.543	0.392	0.395
Mean	0.459	0.473	0.390	0.470
SD	0.008	0.053	0.013	0.160

**Table 11:** Sensitivity analysis for Models A, B, C, and D.

**Neural Network**	**Model A (Output: VA)**			**Model B (Output: CP)**					
	**EM**	**IM**	**VI**	**IN**	**EM**	**IM**	**VI**		
ANN1	0.418	0.311	0.271	0.380	0.296	0.205	0.119		
ANN2	0.366	0.302	0.332	0.248	0.190	0.338	0.225		
ANN3	0.206	0.403	0.391	0.303	0.279	0.179	0.240		
ANN4	0.399	0.291	0.309	0.292	0.386	0.151	0.171		
ANN5	0.362	0.330	0.309	0.325	0.285	0.191	0.199		
ANN6	0.440	0.276	0.284	0.329	0.230	0.290	0.152		
ANN7	0.359	0.304	0.336	0.288	0.278	0.255	0.178		
ANN8	0.420	0.316	0.264	0.264	0.409	0.127	0.200		
ANN9	0.305	0.372	0.323	0.266	0.364	0.211	0.159		
ANN10	0.382	0.355	0.262	0.340	0.361	0.151	0.148		
RI	0.366	0.326	0.308	0.304	0.308	0.210	0.179		
NI (%)	100%	89.071%	84.153%	98.603%	100%	68.161%	58.187%		
**Neural Network**	**Model C (Output: CE)**			**Model D (Output: CIBI)**					
	**IN**	**EM**	**IM**	**VI**	**IN**	**VI**	**VA**	**CP**	**CE**
ANN1	0.364	0.244	0.201	0.191	0.187	0.320	0.287	0.112	0.093
ANN2	0.289	0.340	0.165	0.206	0.140	0.220	0.270	0.211	0.158
ANN3	0.246	0.308	0.200	0.246	0.255	0.298	0.198	0.145	0.103
ANN4	0.401	0.207	0.129	0.263	0.127	0.292	0.304	0.160	0.117
ANN5	0.293	0.290	0.237	0.180	0.191	0.252	0.238	0.156	0.163
ANN6	0.210	0.336	0.325	0.129	0.219	0.274	0.194	0.156	0.156
ANN7	0.423	0.210	0.228	0.139	0.186	0.238	0.277	0.153	0.146
ANN8	0.355	0.274	0.197	0.173	0.189	0.293	0.233	0.135	0.149
ANN9	0.292	0.342	0.093	0.273	0.167	0.301	0.182	0.192	0.159
ANN10	0.213	0.368	0.201	0.217	0.136	0.274	0.263	0.207	0.121
RI	0.309	0.292	0.198	0.202	0.180	0.276	0.245	0.163	0.137
NI (%)	100.000%	94.588%	64.031%	65.360%	65.062%	100.000%	88.559%	58.907%	49.421%

**Table 12:** Comparison between PLS-SEM and ANN results.

PLS-SEM Path	PLS-SEM Path Coefficient	ANN Normalized Relative Importance (%)	Ranking (PLS-SEM)	Ranking (ANN)	Remark
Model A (Output: VA)					
EM→VA	0.220	100%	1	1	Match
IM→VA	0.166	89.071%	2	2	Match
VI→VA	0.160	84.153%	3	3	Match
Model B (Output: CP)					
IN→CP	0.233	98.603%	2	2	Match
EM→CP	0.241	100%	1	1	Match
IM→CP	0.169	68.161%	3	3	Match
VI→CP	0.140	58.187%	4	4	Match
Model C (Output: CE)					
IN→CE	0.190	100.000%	1	1	Match
EM→CE	0.190	94.588%	1	2	/
IM→CE	0.107	64.031%	3	4	/
VI→CE	0.101	65.360%	4	3	/
Model D (Output: CIBI)					
IN→CIBI	0.112	65.062%	5	3	/
VI→CIBI	0.228	100.000%	1	1	Match
VA→CIBI	0.216	88.559%	2	2	Match
CP→CIBI	0.132	58.907%	4	4	Match
CE→CIBI	0.154	49.421%	3	5	/

**Table 13:** Themes identified through grounded theory-informed coding.

NO.	Interview Excerpts	Axial Codes	Selective Codes
1.	“When the digital human introduces a product, I am easily attracted.” “When it mentions discounts or limited-time promotions, I tend to feel tempted and think it would be a pity not to buy now.” “I originally just wanted to browse casually, but the more I watched, the more interested I became.”	-Visual stimulation -Consumption incentive stimulation	Consumer perceived arousal
2.	“When watching the digital human live stream, I feel as if I am actually in the live-streaming room.” “It continuously presents product details, which makes me keep watching for longer.” “The visuals and explanation rhythm are well coordinated, making it easy to become immersed.”	-Immersion in the live-streaming context -Sustained viewing involvement	Immersion
3.	“It explains product functions clearly and appears professional.” “If it explains the ingredients, usage methods, and suitable users, I will have greater trust in the product.” “Some streamers only emphasize ‘cheap’ and ‘buy now,’ whereas digital humans explain products in a more organized way.”	-Professional explanation -Complete product information	Perceived professionalism
4.	“It clearly explains the advantages of the product rather than simply asking me to buy it.” “It explains the price, functions, and current discounts clearly, so I can make my own judgment after listening.” “After hearing its introduction, I feel that buying now may be more appropriate.”	-Product value -Purchase decision guidance	Information persuasiveness
5.	“The usage scenarios it describes are similar to my own situation, so I feel more involved while listening.” “Its tone is friendly and does not feel like simple promotion.” “Sometimes I feel that it understands consumers’ needs quite well.”	-Scenario fit -Affinity	Empathy
6.	“Its facial expressions and movements are relatively natural and do not seem robotic.” “Its tone rises and falls during the explanation, and the pauses are also natural, making the presentation less boring.” “The product presentation is specific, including details and usage methods, so I can remember it more easily.”	-Anthropomorphism -Vivid product presentation	Vividness
7.	“If the digital human looks attractive, I am willing to stop and watch.” “If its makeup, outfit, and overall live-streaming style are well coordinated, it becomes more attractive to me.” “A refined overall visual effect gives me a favorable impression.”	-Appearance design -Coordination of visual style	Visual appeal
8.	“I did not plan to buy at first, but I eventually placed an order.” “When I see a limited-time discount, I feel afraid of missing out and think it may not be worthwhile if I do not buy now.” “At the moment of placing the order, I did not think too much; I was mainly influenced by the live-streaming atmosphere and product explanation.”	-Limited-time discounts -Context-driven purchase	Consumer impulse buying intention
9.	“It can respond to my questions, which gives me a sense of communication.” “Sometimes it continues explaining based on viewers’ comments. For example, if someone asks about usage, it provides further explanation.” “It does not only introduce products one-way; it also guides interaction.”	-Timely response -Interactive participation	Interactivity
10.	“I look at the comment section and click on product links.” “When there are coupons or lottery activities, I want to participate.” “If its explanation is interesting, I will not swipe away immediately and may continue watching what it recommends next.”	-Convenient operation -Promotional activities	Consumer engagement
11.	“It explains with enthusiasm, and I am also influenced by it.” “When the live-streaming room becomes lively and everyone is asking questions or placing orders, I feel like following along.” “Its tone is positive, and when it keeps emphasizing discounts, I feel that buying now seems more worthwhile.”	-Emotional contagion -Group atmosphere influence	Emotional contagion
12	“I do not need to search for information myself because it has already explained the key points.” “After watching the introduction, if I think the product is suitable, I can directly click the link and place an order, which makes the whole process more convenient.” “It helps me quickly judge whether the product is suitable for me and saves time.”	-Convenient information access -Improved purchase efficiency	Perceived convenience

Source: Authors’ own compilation.

**Table 14:** Joint Display of Quantitative and Qualitative Findings.

Quantitative Path	Quantitative Result	Representative Interview Quotations	Integration
IN → CE	Significant	“It responds to my questions, which makes the interaction feel more like a genuine exchange.”; “It does not simply provide information in a one-way manner; it also encourages me to interact.”	Convergence
EM → CP	Significant	“Its tone is warm and friendly, so it does not feel like pure sales promotion.”; “Sometimes, it feels as though it genuinely understands consumers’ needs.”	Partially consistent/Mechanistic complementarity
VI → VA	Significant	“Its facial expressions and movements are natural and do not appear robotic.”; “The product presentation is detailed, showing features and usage methods visually, which makes the information easier to remember.”	Convergence
VA → CIBI	Significant	“The overall visual presentation is polished, which creates a favorable impression.”; “If the digital human is visually appealing, I am more willing to stop and continue watching.”	Conditionality
CP → CIBI	Significant	“When it presents discounts or limited-time offers, I become excited and feel that it would be a pity not to buy immediately.”; “I initially intended to browse casually, but as I continued watching, I gradually felt tempted to make a purchase.”	Convergence
CE → CIBI	Significant	“I read the comments and also click on the product links.”; “When coupons or prize-draw activities are available, I am more willing to participate.”	Conditionality
EM → CIBI	Non-significant	“Its tone is warm and friendly, so it does not feel like pure sales promotion.”; “Sometimes, it feels as though it genuinely understands consumers’ needs.”	Complementarity
IM → CIBI	Non-significant	“When watching a digital human livestream, it feels as though I am actually in a livestreaming room.”; “The visuals and pacing of the explanation work well together, making it easy to become immersed as I continue watching.”	Complementarity

**Table 15:** Research findings and insights.

Research Dimensions	Key Findings	Theoretical Implications	Management Implications
DHA cues and consumer experience	Within the proposed model, empathy (EM) exhibits a relatively strong positive association with consumer-perceived arousal (CP), while CP is significantly and positively associated with CIBI.	By contextualizing the S-O-R framework within the EC-DHA livestreaming setting, this study identifies differentiated relationships between specific EC-DHA cues and consumer experiential states. Among these relationships, the association between empathy (EM) and consumer-perceived arousal (CP) is particularly pronounced.	In practical terms, platforms may optimize digital humans’ appearance, interaction style, emotional expression, and product information presentation according to specific livestreaming contexts to enhance consumers’ livestream shopping experiences.
Consumer experience and impulse buying intention	Visual appeal (VA), consumer-perceived arousal (CP), and consumer engagement (CE) are all significantly and positively associated with CIBI, with VA exhibiting the relatively largest path coefficient.	This finding enriches impulse buying theory by emphasizing the importance of visual and emotional experience in DHA-mediated shopping environments.	Platforms may consider visual presentation, interaction guidance, emotional expression, and information delivery as key reference factors for optimizing user experience, rather than treating them as deterministic design strategies that directly drive purchasing behavior.
Quantitative research: PLS-SEM and ANN	The combined use of PLS-SEM and ANN provided complementary validation. Models using VA, CP, and CE as output variables showed generally consistent results across the two methods, while also revealing differences in path sensitivity.	This study constructs an integrated methodological framework that combines quantitative path significance with nonlinear variable importance, thereby improving the robustness of model validation.	Platforms can establish a two-layer evaluation dashboard, using SEM to identify structural paths and ANN to quantify predictive influence, thereby improving the interpretability and transparency of consumer behavior assessment.
Qualitative Study: Grounded Theory-Informed Analysis	The qualitative findings indicate that DHA visual appeal (VA), vividness (VI), and related experiential factors play key roles in shaping consumers’ impulse buying judgments and purchase intention formation.	This finding provides a user-centered explanation of how DHAs stimulate consumer impulse buying intention in e-commerce live-streaming contexts.	An integrated design strategy that coordinates visual appearance, emotional communication, information delivery, and interaction guidance may help enhance consumer experience and strengthen impulse buying intention.

Source: Authors’ own compilation.

## Data Availability

All data generated or analyzed during this study are included in this article. The raw data are available from the corresponding author upon reasonable request.

## Appendix A

**Table A1:** Distribution of population characteristics.

Variable	Options	Frequency	Percentage
Gender	Male	212	45.40%
	Female	255	54.60%
Age	Under 18	0	0.00%
	18–25	177	37.90%
	26–35	166	35.55%
	36–45	78	16.70%
	46–55	34	7.28%
	56 and above	12	2.57%
Degree	High school or below	34	7.28%
	Junior college	79	16.92%
	Bachelor’s Degree	231	49.46%
	Master’s Degree	103	22.06%
	Doctoral Degree and above	20	4.28%
Occupation	Student	126	26.98%
	Company employee	207	44.33%
	Civil servant/Public institution employee	48	10.28%
	Self-employed	36	7.71%
	Freelancer	31	6.64%
	Other	19	4.07%

**Table A2:** Basic information of the interviewee.

Participant	Region	Gender	Age	Occupation
P1	Wuhan	Female	54	Teacher
P2	Wuhan	Male	56	Support staff
P3	Wenzhou	Female	21	Student
P4	Tianjin	Male	23	Student
P5	South Korea	Female	27	New media operator
P6	Zhengzhou	Male	31	Engineer
P7	Zhaoqing	Female	36	Teacher
P8	Wuhan	Male	42	Self-employed business owner
P9	Changsha	Female	46	Nurse
P10	Ningbo	Male	66	Retiree
P11	Wenzhou	Female	19	Student
P12	Chongqing	Male	25	Food delivery rider
P13	South Korea	Female	29	Aesthetic physician
P14	Zhengzhou	Male	34	Fitness coach
P15	Guangzhou	Female	38	Bank clerk
P16	Wuhan	Male	41	Ride-hailing driver
P17	Zhuzhou	Female	48	Community worker
P18	Shenzhen	Male	52	Technical staff
P19	South Korea	Female	60	Retired teacher
P20	Wenzhou	Male	24	Student
P21	Changsha	Female	30	Student
